# Spatial organization, chromatin accessibility and gene-regulatory programs defining mouse sensory neurons

**DOI:** 10.1038/s42003-025-08315-1

**Published:** 2025-06-11

**Authors:** Doris Krauter, Jussi Kupari, Dmitry Usoskin, Jie Su, Yizhou Hu, Ming-Dong Zhang, Patrik Ernfors

**Affiliations:** Department of Medical Biochemistry and Biophysics, Division of Molecular Neurobiology, Karolinska Institutet, Stockholm, Sweden

**Keywords:** Pain, Somatic system

## Abstract

Heterogeneity among somatosensory neurons is necessary for internal and external sensation. Precise patterns of gene transcription orchestrated through enhancer activation maintain heterogeneity. Thus, high-resolution cell type classification, chromatin accessibility and its relation to enhancer activation can explain the governing principles for sensory neuron heterogeneity. Here, we present an integrated atlas from published high-quality scRNA-seq datasets and resequencing the dorsal root ganglion, including over 44,000 neurons. MERSCOPE spatial transcriptomics confirms cell types in situ, including previously unrecognized neuronal types, and a spatial zonation of both neurons and non-neuronal cells. We present a cell type specific open chromatin atlas revealing enhancer driven regulons and gene-regulatory networks organized into co-regulated gene-programs that together define sensory neuron diversity. Cell type complexity is shown to be generated by layered co-regulated transcriptional modules representing shared functions across different scales of the neuronal type hierarchy with cell type specific contribution as the exception.

## Abstract (web summary)

Single cell RNA-sequencing, spatial transcriptomics and chromatin analyses of sensory neurons provide insights into cell type organization of the dorsal root ganglion and the enhancer-driven co-regulated gene programs generating heterogeneity.

## Introduction

The contribution of a population of somatosensory neurons for a sensation relies on its functional properties, which are determined by the molecular makeup. Thus, classifying dorsal root ganglion (DRG) neurons based on molecular features is essential to understanding the neural principles for sensing the world within and around us. Single-cell RNA sequencing (scRNA-seq) presents a tangible way to define cell types and states. Mouse dorsal root ganglion sensory neurons have been extensively studied using scRNA-seq and these studies have revealed the existence of more than a dozen different subtypes enabling predictions and experimental evidence on function based on gene expression^1–7^. While the different scRNA-seq studies largely overlap in terms of the identified neuronal subtypes, there are variations across the studies in relation to the resolution of cell type identification, the sampling size and quality of the data, for example depth of sequencing and satellite cell contamination. Thus, while each dataset has its own strengths, they all also have weaknesses. Furthermore, some studies used single nucleus RNA-sequencing (snRNA-seq) while others used scRNA-seq. Cross-study variability is also created as cytosolic RNA differs from nuclear RNA in terms of abundance as well at the level of transcript types and gene expression levels^8^. In addition, the datasets were generated using partly different technological platforms, adding cross-platform variability. However, high-quality integration can allow the identification of recurring cell types across studies, increase resolution of cell type identification, decrease noise and increase faithfulness of identified gene expression properties that are necessary for predicting function.

At the core of the process that regulates diversity, and function is the transcriptional circuitry. To investigate the mechanisms underlying the transcriptional features of different cell types, it is highly desirable to not only examine gene expression but also epigenetic underpinnings such as chromatin accessibility^9^. Joint profiling of chromatin accessibility and gene expression in individual cells can explain how expressed gene regulatory networks are driven by enhancer activity^10^ and how such enhancer gene regulatory networks are organized into a multi-scale hierarchy of functional units across individual cell types^11^.

A weakness of scRNA-seq is that the tissue dissociation process leads to the loss of spatial context. Spatial transcriptomics emerged to address these challenges and to delineate cellular functions, cellular states and their organization within the tissue^12^. Sensory neurons arise from neural crest cells delaminating from the neural tube and migrating ventrally. At the time of delamination, neural crest cells lose their tissue organization, and hence, this might suggest a random spatial arrangement of neuronal types in the ganglion. However, tissue organization and thereby instructive signaling changes during the period of migration and neurogenesis^13^. Furthermore, upon commitment to a neuronal fate, sensory neuron progenitor cells further divide a few times^14^. Thus, because neuronal types can be affected by both the timing of neurogenesis and inherent properties of progenitor cell division, organization of the molecularly defined neurons in the DRG might not be random, a conjecture supported not the least by studies on the chick^15^.

To gain a deeper understanding of tissue organization, the heterogeneity and how it is built by engaging transcriptional programs, we generated a high-quality integrated scRNA-seq atlas of cells in the DRG encompassing over 44,000 neurons and non-neuronal cells, determined the spatial organization of neuronal and non-neuronal cell types and identified accessible chromatin to explain the logic for enhancer activity that generates diversity across subtypes of neurons.

## Results

### DRG neurons and their spatial organization

An integrated transcriptomic atlas offers the unique opportunity to use consistent cell type labels across independently sampled datasets so that robust analyses can be conducted at varying levels of specificity in the cell type hierarchy. As the nucleus contains only a fraction of cellular RNA content and cross platform data can generate technical variability, we focused on five whole cell sequencing studies using the 10x Genomics Chromium 3’ scRNA-seq platform (named after their first authors, “Finno”^1^, “Sharma”^2^, “Wang”^3^, “Zeisel”^4^, “Zhang”^5^, “Su”^16^) with varying depth of sequencing and furthermore conducted new high-quality re-sequencing from CD1 mice (“CD1”) (Supplementary Fig. S1a).

**Fig. 1:**
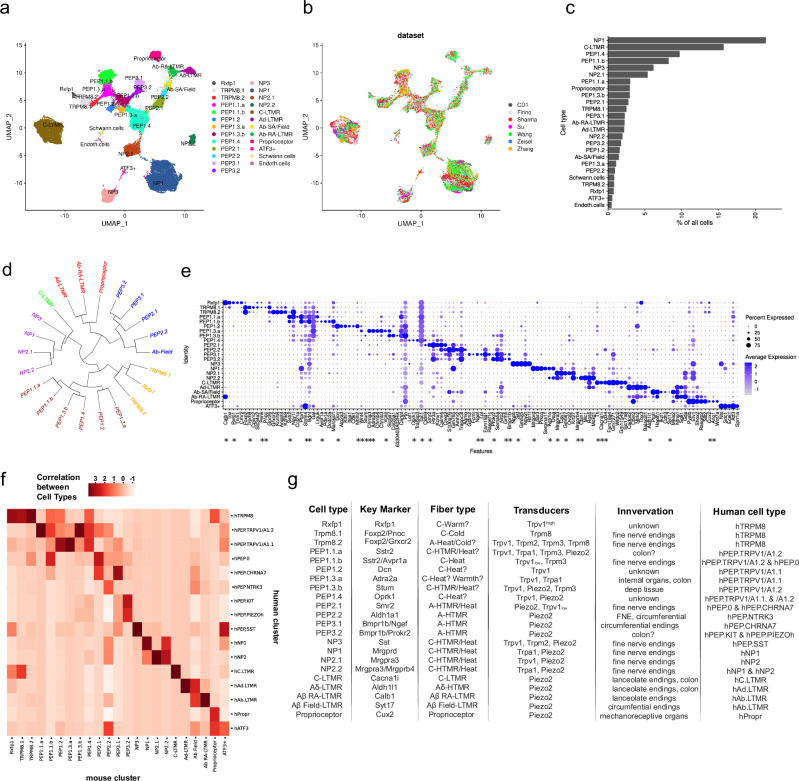
Integrated atlas of mouse DRG neurons. **a** UMAP of cell populations defined in the integrated atlas, including ATF3+ cells, Schwann, and endothelial cells that coalesced from the original datasets. **b** UMAP showing the original datasets to the cell populations. **c** Bar plot of the percentage contribution of each cell-type in the integrated atlas. **d** Hierarchical relationships of the defined neuron types (excluding the ATF3+ cells) in the integrated atlas shown in a fan-shaped dendrogram. **e** Dot plot of top marker genes for each of the neuron types in the integrated atlas. Genes marked with asterisks were used as markers for the MERSCOPE data in further studies. **f** Heatmap of neural network learning of the integrated mouse atlas and human DRG scRNA sequencing data. Color intensity shows the strength of the correlation between cell types. **g** Summary table of the identified mouse neuronal cell types, their key marker, fiber type, transducers, innervation target^17–20,61^, and corresponding human cell type^21^. Question mark indicates the assumed subtype in the split that innervates the colon among *Sstr2* and *Bmpr1b* expressing neurons). FNE fine nerve endings.

After quality control for each dataset (see methods), the remaining 47,121 cells from the original datasets were subjected to supervised neuron type labeling using each individual dataset as source for a classifier. In essence, each individual dataset was used to create a model defining the gene expression patterns of each individual cell type defined in the data. These dataset specified gene expression patterns were then used to label each cell in the other datasets. As potential undiscovered heterogeneity would be “hidden” among previously annotated clusters, we kept only cells which were assigned to corresponding clusters according to each individual dataset classification (Su, Zeisel, Sharma and Wang). This criterion was used for all clusters except A fiber-type low threshold mechanoreceptors (LTMRs) since assignments within these clusters were not reliable in some of the used datasets. 45,382 neurons passed this filtering step and the resulting dataset was subjected to unbiased clustering where gene expression patterns in the full data were learned without outside input. The final atlas consisted of 44,274 neurons that clustered into 23 transcriptionally distinct compartments including ATF3^+^ neurons, averaging ~4300 detected genes and 27,000 unique molecules per cell (Fig. 1a). In addition, the dataset included 379 satellite and 218 endothelial contaminating cells. Cells from the different original datasets contributed to each cell type in the final atlas; however, two datasets (“Su” and “CD1”) had little contribution to the large, myelinated cell types (Supplementary Fig. S1b). Contribution to cell numbers in the full atlas ranged between 3% and 32% among the datasets (Supplementary Fig. S1c). Contribution of identified neuronal types in the integrated atlas to each original dataset revealed which previous clusters had contained unrecognized heterogeneity and furthermore confirmed that the atlas contained robust and reproducible populations of DRG neurons (Fig. 1b, Supplementary S1d). Probabilistic neural network learning revealed a learning curve of >95%, high robustness, and accuracy but low cross-neuron prediction scores in the trained module (Supplementary Fig. S2a–c). Quantifying the contribution of sequenced cells to molecularly defined cell types showed that some neuronal types contributed close to 20% of the sensory neurons in the DRG, while most were rare and represented <5% of the neurons (Fig. 1c). Hierarchical clustering of the defined neuronal types showed three main branches separating the neurons to: PEP1 C-fiber nociceptors and other likely temperature sensing C-fiber types; NP C-fiber types and C-LTMRs; myelinated LTMRs, Aδ, PEP2 and PEP3 types (Fig. 1d). We observed that PEP2.2 showed unexpected close hierarchical relation to Aβ Field-LTMRs and C-LTMRs to NP-class neurons. All 23 neuronal types were defined by a combination of marker genes of which many were type-specific (Fig. 1e, Supplementary Table S1). Notably, all previously identified neuronal types were confirmed; however, unrecognized heterogeneity within cold-sensing TRPM8 neurons, PEP1.1 and PEP1.3 C-fiber types and PEP2 and PEP3 A-fiber high threshold mechanoreceptor (HTMR) types were found. When heterogeneity was found within a neuron previously annotated as X1, the new clusters were named X1.1 and X1.2 and clusters that in previous studies had already been split into subclusters (for example, X3.1) with further heterogeneity were named X3.1.a and X3.1.b. In this way, the origin of the cells contributing to the new clusters could be related to previously published annotations. Thus, TRPM8 neurons split into TRPM8.1^*Foxp2/Pnoc*^ and TRPM8.2^*Foxp2/Grxcr2*^; PEP1.1 split into PEP1.1.a^*Sstr2*^ and PEP1.1.b^*Sstr2/Avpr1a*^; PEP1.3 into PEP1.3.a^*Adra2a*^ and PEP1.3.b^*Stum*^, PEP2 into PEP2.1^*Smr2*^ and PEP2.2^*Aldh1a1*^; and PEP3 into PEP3.1^*Bmpr1b/Ngef*^ and PEP3.2^*Bmpr1b/Prokr2*^; NP2 into NP2.1^*Mrgpra3*^ and NP2.2^*Mrgpra3/Mrgprb4*^. Although this study was not designed for identifying sex differences, we used a machine learning model to predict cell sex of each individual cell in the atlas (see the “Methods” section). Using these predicted labels for the sex of each cell, we analyzed the proportions in the different cell types between males and females and found no statistical difference in the proportion of any of the neuronal types (Supplementary Fig. S3). Markers of each of the different splits were compared to each other and to the closest third-related neuronal cell type to provide insights into shared and divergent molecular features of the previously unnoticed heterogeneity (Supplementary Fig. S4a–e).

Among the neuronal types, Rxfp1 neurons expressed much higher levels of *Trpv1* than any other neuron population in the DRG (Supplementary Fig. 4f), indicating responsiveness to innocuous warmth stimuli. Interestingly, while TRPM8.1 and TRPM8.2 populations shared several previously identified markers such as *Foxp2*, *Cdh8*, and *Gpr26* they also contained a range of uniquely expressed genes including transducers and neuropeptides (Supplementary Fig. S4a). While TRPM8.1 contained higher levels *Trpm8* and the neuropeptides *Pnoc* and *Penk*, TRPM8.2 neurons expressed lower levels of *Trpm8* as well as the heat-responsive ion channels *Trpv1* and *Trpm3* and warmth-activated ion channel *Trpm2* (Supplementary Fig. S4a and f). Thus, TRPM8.1 might respond to cooling stimuli while TRPM8.2 to thermal stimuli in a range of cold and heat temperatures. PEP1.1.a and PEP1.1.b shared previously identified markers *Sstr2*, *Ndnf*, *Caln1*; however, PEP1.1.a uniquely expressed for example the vasopressin receptor *Avpr1a*, perhaps indicating interoception, while PEP1.1.b expressed *Tgfb1* suggesting potential roles in neuroimmune interactions (Supplementary Fig. S4b). PEP1.3.a and PEP1.3.b shared previously identified markers such as *Kit* and *Gal*, but the former expressed the molecular identifiers for silent C-fiber nociceptors, *Chrna3*^17^, *Adra2a*^18^ along with *Trpa1*, while PEP1.3b uniquely expressed *Piezo2*, *Prokr2*, *Stum* and several other markers (Supplementary Fig. S4c and f). Thus, while both cell types innervate deep tissues^17–19^, they likely display unique response profiles to chemical and mechanical stimuli. The *Piezo2* expressing Aδ-HTMR PEP2 subtypes shared several markers but were different in terms of *Trpv1* expression in PEP2.1 but not in PEP2.2 (Supplementary Fig. S4d and f). Furthermore, the previously used marker to identify this population, *Smr2*^18^, was only expressed by PEP2.1 while PEP2.2 expressed *Cd34*, *Aldh1a1*, *Ntrk3*, and several other genes (Supplementary Fig. S4d). Consistent with the hierarchical relationship to Aβ Field-LTMRs, PEP2.2 shared the expression of many genes while at the same time expressing *Scn10a* (Nav1.8) and other nociceptor-specific genes. The two PEP3 subtypes of neurons both expressed previously used markers *Kit*, *Bmpr1b* along with several other shared markers; however, they also contained unique patterns of gene expression, including *Ngef* and *Lpl* in PEP3.1 and *Prokr2* and *Prlr* in PEP3.2 (Supplementary Fig. S4e). Based on this, PEP3.1 may represent the superficially innervating circumferential Aδ-HTMR endings innervating hair follicles and is known to be involved in a hair pull pain in both mouse and human^20^, while the expression of *Prokr2* in PEP3.2 indicated a bias towards internal innervation of these Aδ-HTMR neurons. Probabilistic neural network learning was used in cross-species comparison between the mouse and a human dataset^21^ (Fig. 1f, Supplementary Fig. S2d, e). In previous analyses without sub-type splits of PEP1.1 and PEP1.3, these neurons had low homology to the human PEP C-fibers. The new mouse subtype splits and/or improved learning using the integrated atlas revealed a relatively high correlation between mouse PEP1.1.a, PEP1.2, PEP1.3.a, and PEP1.4 to human hPEP.TRPV1/A1.1 and hPEP.TRPV1/A1.2 neurons (Fig. 1f). Figure 1g summarizes the identified neuronal populations, expression of markers and transducers, and how these relate to what is known in the literature based on key marker expression.

**Fig. 2:**
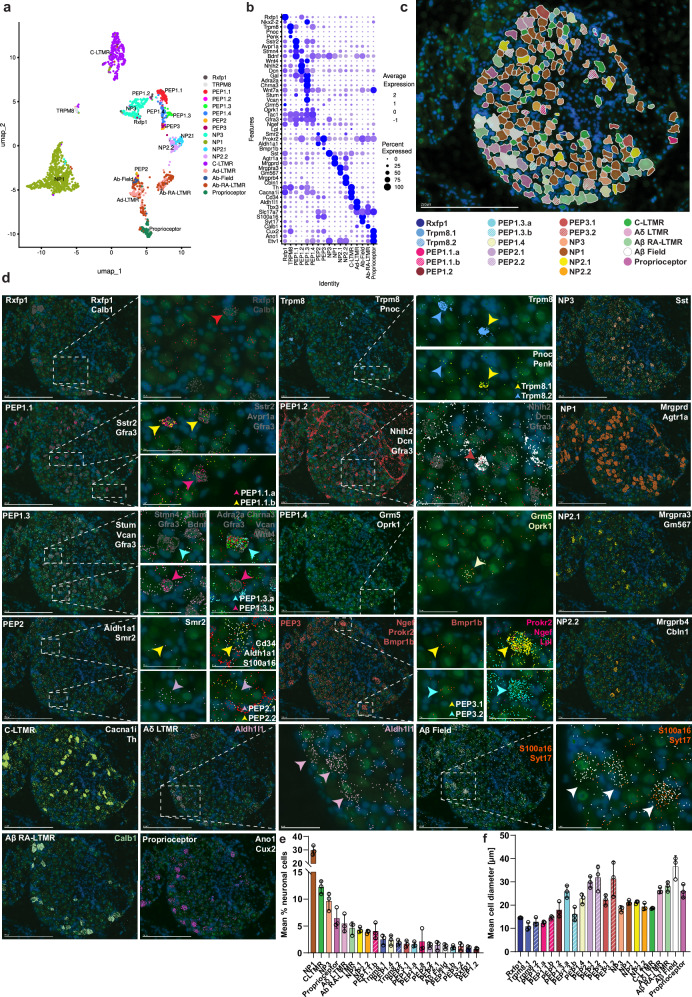
Spatial organization of neurons in the mouse DRG. **a** UMAP showing the cluster annotation of 1857 neurons from MERSCOPE spatial transcriptomics. **b** Dot plot depicting the marker genes for each neuron cell type identified in the MERSCOPE cell clustering. **c** Example section of a DRG with manually color-assigned neuronal cell types. **d** Example images of MERSCOPE for marker genes expressed in each neuronal cell type identified in the integrated atlas. Arrowheads show assigned neurons, e.g. top row, middle: Trpm8.1 neurons (yellow arrowhead) express *Trpm8* (blue), *Pnoc* (yellow), and *Penk* (green) while Trpm8.2 neurons only express *Trpm8* (blue arrowhead). Scale bar is 250 µm in whole DRG section images and 50 µm in insets. **e** Mean percentage of neuron cell types identified in DRG sections and **f** mean cell diameter of neuron cell types in the DRG. 3 DRG sections were annotated and quantified. Bar graphs in **e**, **f** show the mean and error bars represent the standard error of the mean.

MERFISH is a technology based on single-molecule fluorescence in-situ hybridization (smFISH), where fluorescently tagged oligo probes are used to label RNA transcripts of interest, allowing the simultaneous spatial expression profiling of up to thousands of individual genes within intact tissue samples while preserving their spatial context. We used the MERSCOPE (MERFISH) spatial transcriptomics platform on mouse DRGs with a set of 300 probes (Supplementary Table S2) including variable genes in the different subtypes of DRG neurons. Image segmentation to partition out proper cell profiles from the microscopic images was carried out with Cellpose2.0^22^ using a “human-in-the-loop” approach where an investigator first selects a set of true cell profiles from a sample of images and this information is then used to train a model to learn cell profiles from unseen data. After unsupervised clustering of the total 9531 cells, the neurons were labeled using a machine-learning model built on a subset of the integrated atlas with additional non-neuronal data (see the “Methods” section). Learning the cell types based on this model resulted in high-confidence labeling of 1857 neurons, although with lower resolution than in the integrated atlas when performing unbiased clustering (Fig. 2a); however, identification of unique transcriptional features within each main neuron type enabled further subdivision of the neurons to all types identified in the atlas (Fig. 2b–d, Supplementary Fig. S5).

Quantitative analysis of the in-situ contributions from different neuron types as a percentage of all neurons revealed that the scRNA-seq data skewed the predicted representations to some degree (Fig. 2e). The analysis further revealed NP1, C-LTMRs, and NP3 neurons to be most abundant while most neuronal populations often represented only a few percent of the DRG neurons. Quantification of mean cell diameter revealed Aβ Field-LTMRs as the largest type and the remaining A-LTMRs, proprioceptors, Aδ fiber-HTMRs such as PEP2.1, PEP2.2, PEP3.2 and unexpectedly the C-fiber PEP1.3.a and PEP1.4 as other large size neurons (Fig. 2f).

**Fig. 3:**
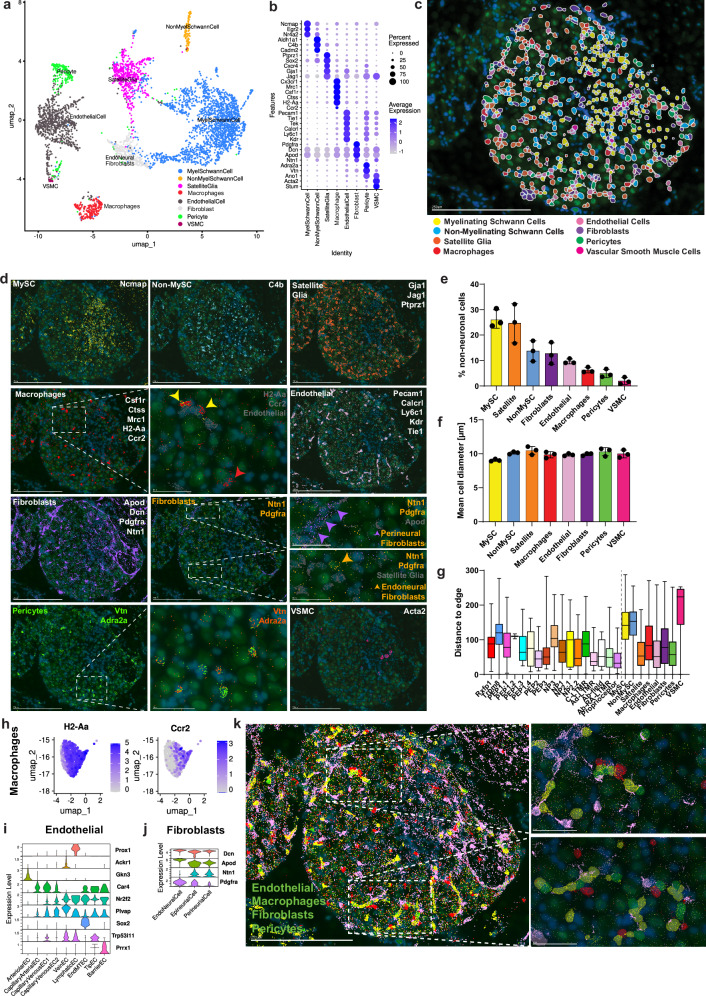
Spatial organization of non-neuronal cell types in the mouse DRG. **a** UMAP showing the cluster annotation of 4036 non-neuronal cells from MERSCOPE spatial transcriptomics. **b** Dot plot depicting the marker genes for each non-neuronal cell type identified in the MERSCOPE cell clustering. **c** Example section of a DRG with manually color-assigned non-neuronal cell types. **d** Example images of MERSCOPE for marker genes expressed in each non-neuronal cell type. Yellow arrowheads show *Ccr2*+ subpopulation of Macrophages and red arrowheads *Ccr2*- population (second row, middle panel). Purple arrowheads depict perineural fibroblasts and orange arrowheads endoneural fibroblasts (third row, right panel). Scale bar is 250 µm in whole DRG section images and 50 µm in insets. **e** Mean percentage of non-neuronal cell types identified in DRG sections and **f** mean cell diameter of neuron cell types in the DRG. 3 DRG sections were annotated and quantified. Bar graphs in **e**, **f** show the mean and error bars represent the standard error of the mean. **g** Boxplot showing the distance of neuronal and non-neuronal cell types to the edge of the DRG section. *n* = 6 sections. Boxplot upper edge, center line, and lower edge define third quartile, media,n and second quartile (respectively). The upper and lower whiskers define minimum and maximum values (respectively). **h** UMAPs showing expression for selected genes in endoneurial macrophages. **i** Violin plots showing expression of selected genes in the untreated endothelial cell types. **j** Violin plots showing expression of selected genes in the untreated fibroblast cell types. **k** Example section of the DRG with a merged marker for endothelial (yellow), macrophages (red), fibroblasts (pink), and pericytes (green). On the right are close-ups showing the corresponding assigned cell types from the clustering of non-neuronal cells. Scale bar is 250 µm in the whole DRG section image and 50 µm in insets.

To uncover the spatial organization of neurons we analyzed the distribution within the ganglion, measuring distance to the edge of the ganglion (Supplementary Fig. S6a). This revealed a robust organization of the neurons with A-LTMRs, PEP2 and PEP3 neurons at the outer perimeter, an intermediate and central perimeter containing most neuronal types with TRPM8 and NP3 neurons being most distant from the outer edge (Fig. 3g). To establish whether there is a spatial context of neuron types relative to each other, we quantified pairwise spatial proximity as a neighbor score, with 1 indicating maximum co-localization and 0 indicating no spatial overlap. This revealed that neurons of the same type and those with closer hierarchical relationships displayed closer proximity to each other than to more distantly related types (Supplementary Fig. S6c).

### Non-neuronal cells in the DRG and their spatial organization

Spatial organization can affect oxygenation, nutrient supply as well as neuroimmune interactions. A total of 4036 cells were labeled with high confidence as different types of non-neuronal cells from the MERSCOPE data using the supervised learning strategy described above. Predicted identities for the eight non-neuronal clusters included myelinating and non-myelinating Schwann cells, satellite glial cells, endothelial and endoneural fibroblasts, pericytes, vascular smooth muscle cells, and macrophages (Fig. 3a). All labeled types showed a clear cell type-specific gene expression pattern providing high-confidence type assignment (Fig. 3b and c). While myelinating Schwann cells were enriched at the nerve tract, non-myelinating Schwann cells were enriched at the intermediate and central perimeter containing most neuronal types. Satellite glial cells were most abundant in the outer perimeter containing A-LTMRs with moderate abundance in the intermediate zone and low cell abundance in the nerve tract (Fig. 3d). Macrophages were scattered throughout the ganglion and were characterized by expression of *Cx3cr1*, *Csf1r*, *Ctss*, and H2-Aa as well as the phagocytic receptor *Mrc1*. One macrophage subtype expressed *Ccr2* present in tissue-resident macrophages^23^ (Fig. 3). *Ccr2*^+^ macrophages were uniquely associated with endothelial cells (Fig. 3d and k), consistent with a recent study^24^. All fibroblasts were characterized by expression of the known markers *Apod*, and *Dcn*^25^; however, expression of the axon guidance molecule netrin 1 encoded by *Ntn1* defined perineural and epineural fibroblasts lining the DRG and large nerve bundles, while *Pdgfra* was expressed exclusively by endoneural (interstitial) fibroblasts scattered within the ganglion and close to satellite glial cells (Fig. 3d and j). Apolipoprotein D encoded by *Apod* is expressed in nonproliferating quiescent fibroblasts^26,27^. *Vtn* (encoding the extracellular matrix protein vitronectin), *Pdgfrb* and *Adra2a* were expressed in pericytes associated with vascular endothelial cells (Fig. 1d, k), as has been described for CNS pericytes. Vitronectin is secreted by central nervous system pericytes involved in blood–CNS barrier function^28^. Finally, we found *Acta2* + (encoding alpha-2 smooth muscle actin) cells which distinguish vascular smooth muscle cells (VSMCs) that are the typical contractile cells associated with parenchymal arterioles.

Quantification of the relative abundance of the different non-neuronal cell types and neurons suggested a rich microenvironment created by the different resident cell types within the ganglion and a uniform size across the different non-neuronal cells in the DRG (Fig. 3e and f). Analysis of the spatial localization showed myelinating Schwann cells, non-myelinating Schwann cells, and VSMCs had the greatest mean distance to the edge of the ganglion (Fig. 3g). Visual inspection suggested that some non-neuronal cells displayed a non-random organization within the ganglion. Like for neurons, we quantitatively analyzed the proximity of non-neuronal cells to other non-neuronal cells to obtain detailed information on the cell–cell organization (Supplementary Fig. S6d and e). We were particularly interested in the cellular organization of capillaries, because of the capacity of high molecular weight molecules to leak by endothelial cell caveolar transcytosis and fenestrae and engage macrophages causing an inflammatory response^24,29,30^. Analysis of the scRNA-seq data revealed nine types of endothelial cells (ECs) in the DRG with unique patterns of gene expression (Supplementary Fig. S7). The main types as defined by marker expression in Kalucka et al^31^. were arteriolar, postcapillary venules (vein), lymphatic, and three types of capillary endothelial cells forming the microvascular bed of the DRG (Supplementary Fig. S7b). In addition, three rare types with more unclear molecular signatures were classified as endothelial-mesenchymal transition ECs (EndMT-ECs, *Sox2*+^32^), Tip EC (*Trp53i11*^33^) and barrier EC (*Prrx1*^34^). The three capillary endothelial cells were a continuum and expression of arterial and venous capillary markers revealed a graded inverse relationship of expression in arterial-capillary EC, capillary-venous EC1, and capillary-venous EC2 types, including *Plvap* peaking in capillary-venous EC2 and vein ECs. Plasmalemmal vesicle-associated protein-1 encoded by *Plvap* is restricted to caveolae and forms fenestral diaphragms, conferring the microvascular permeability^35^. Endothelium, pericyte, macrophage, and fibroblasts are closely associated with the DRG^24^. Analysis of spatial transcriptomics revealed such perivascular neuro-immune cellular units in capillaries to consist of endothelial cells associated with *Vtn*^+^ pericytes, the *Ccr2*^+^ macrophages and *Dcn*^+^/*Ntn*^−^ endoneural fibroblasts (Fig. 3j) within the DRG (Fig. 3k).

### Accessible chromatin and enhancers-driven expression of target genes defining sensory neurons

Assay for Transposase-Accessible Chromatin with high-throughput sequencing (ATAC-seq) is a sequencing technique that maps regions of accessible chromatin across the genome using a transposase that cuts DNA areas where chromatin is less condensed. These regions often correspond to active regulatory elements like promoters and enhancers allowing, especially in combination with gene expression information, the identification genome-wide patterns in gene regulation and cell-type-specific regulatory landscapes. We generated a single-nucleus RNA- and ATAC-seq multiome atlas of DRG sensory neurons by co-profiling the epigenome and transcriptome of 4714 neurons. The data showed on average ~4800/29,000 unique genes/molecules and ~20,000/15,000 highly accessible areas (peaks)/DNA fragments per nucleus along with good levels for other quality metrics (Supplementary Fig. S8a–c). Two-thirds of the peaks fell in regions of the genome not overlapping known candidate cis-regulatory elements defined by ENCODE, while ~24% coincided with regulatory regions controlling genes more than 2000kb away. The remaining few percent overlapped regulatory regions close to genes, promoters, or DNA-sensitive sites. ~90% of the peaks were situated within introns or intergenic regions and the rest were in promoters, exons, transcription termination sites, and 3’ and 5’ untranslated regions (Supplementary Fig. S8d). Most regions were annotated to one or two genes and similarly, most genes were connected to one to few regions, yet some genes had up to 300+ annotated regions (Supplementary Fig. S8e).

**Fig. 4:**
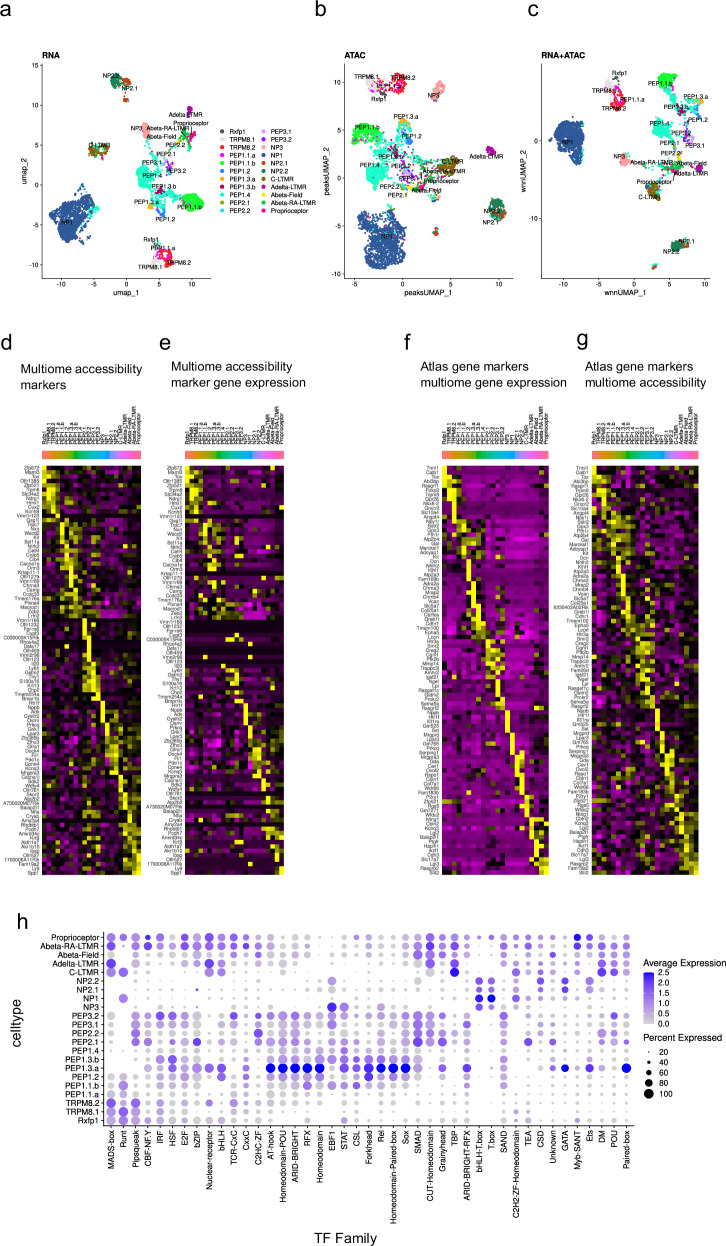
Multiome-seq of the mouse DRG neurons. **a** UMAP presentation of the mouse DRG single-nuclei data based on the RNA signal, annotated according to the integrated atlas neuron types. **b** UMAP of the mouse DRG multiome data clustered based on ATAC signal. **c** Weighted nearest neighbors based UMAP of the mouse DRG multiome data. **d** Heatmap showing mouse DRG marker genes based on gene body chromatin accessibility signal. **e** Gene expression heatmap of accessibility marker genes from (**d**). **f** Heatmap neuron type marker genes (defined in the integrated atlas) shown in the multiome RNA data. **g** Heatmap of gene body chromatin accessibility of the marker genes in (**f**). **h** Dot plot showing the top transcription factor motifs and their families for each DRG neuron type.

After unbiased clustering using the transcriptome data, the cell type identities for individual nuclei were determined with a supervised labeling strategy based on a classifier model built on a subset of the integrated atlas (see the “Methods” section) (Fig. 4a). Unsupervised clustering and dimensional reduction to a UMAP using the chromatin accessibility data revealed clear separation of the cell types that had been labeled using the transcriptome data (Fig. 4b). A weighted nearest neighbors approach combining both the snRNA- and snATAC-seq corroborated the single modality analyses implying a good quality dataset (Fig. 4c).

We then aggregated the signal from sequenced fragments spanning the transcription start site and gene body for each cell type in the ATAC-seq data to find marker genes with the highest accessibility (Supplementary Table S3). We found an overall strong correlation between individual gene accessibility and gene expression of downstream genes, with many known cell type markers showing high accessibility in the appropriate types, for example, *Trpm8* in TRPM8.1 and TRPM8.2; *Ntrk2* in Aδ-LTMR; *Chrna3* in PEP1.3.a; *S100a16* in PEP2.3 and Aβ Field-LTMRs; *Nbbp* and *Osmr* in NP3; *Lpar3* in NP1; *Mrgpra3* in NP2.1 and NP2.2 and *Cacna1i* in C-LTMRs. However, some genes showed accessibility without corresponding gene expression, indicating open chromatin regions without transcription (Fig. 4d and e). To further validate these observations, we examined gene expression and chromatin accessibility in the multiome data for markers defined in the scRNA-seq integrated atlas, seeing a similarly strong correlation between gene expression and accessible chromatin, yet sometimes more widespread chromatin accessibility than gene expression (Fig. 4f and g). We proceeded to investigate the variability of binding motif enrichment for transcription factors (TF) families in the sequenced fragments using ChromVar and a curated database of 884 mouse motifs (Fig. 4h, Supplementary Table S4). ChromVar calculates “accessibility deviation scores” for transcription factor motifs by comparing the observed accessibility at binding sites to a background of similar regions, accounting for biases like GC content and read depth. This approach helps identify which transcription factors are potentially active in specific cell types based on the accessibility of their DNA binding sites. We found a cell-type enrichment of motifs for the MADS-box family, Nuclear receptor family motifs, Runt family motifs, Homeodomain family motifs, POU motifs, CUT-Homeodomain motifs, T-box motifs, Ets family motifs, and more.

**Fig. 5:**
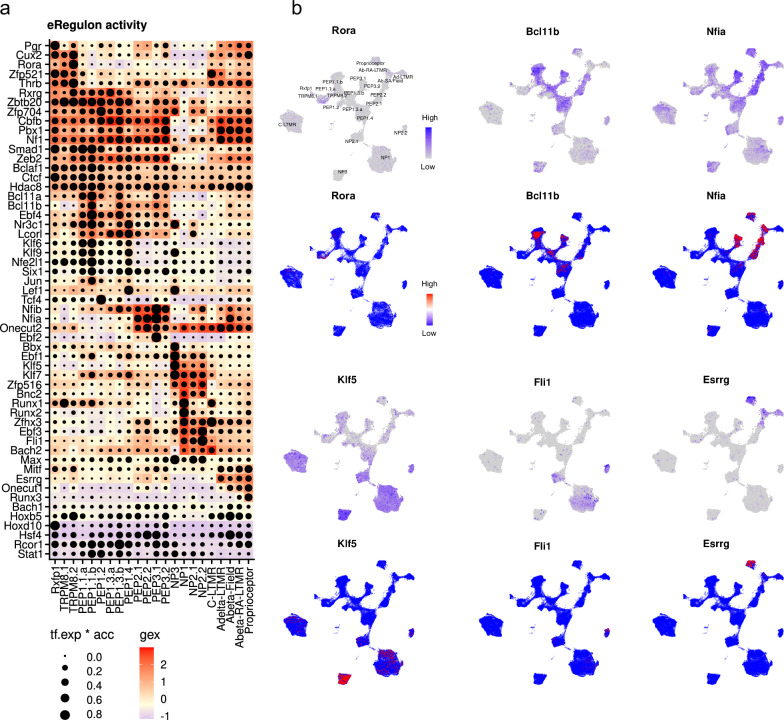
eRegulon activity across mouse DRG neuron types. **a** Dot plot showing the activity of selected eRegulons in the mouse DRG multiome data (the full plot is shown in Fig. S7c). Dot size represents the product of transcription factor (TF) expression (% of cells expressing the gene in the cell type) and chromatin accessibility of the combined group of regions controlled by the TF (chromatin regulon). Color represents the gene expression score of the combined group of genes under the control of the chromatin regulon (gene regulon). **b** UMAP presentations of the gene regulon activity of example eRegulons from **a** in the integrated atlas (all regulons are shown in Fig. S7).

Simultaneous profiling for gene expression and chromatin accessibility makes it possible to investigate gene regulation in combination with enhancer activity. To accomplish this, we used the SCENIC+ pipeline^10^. Briefly, SCENIC+ allows the integration of gene expression and chromatin accessibility data to infer gene regulatory networks in single cells. On top of transcription factors and their target genes, SCENIC+ incorporates information from regulatory DNA elements from ATAC-seq providing a more comprehensive view of gene regulation. The output of SCENIC+ is a set of TF-region-gene combinations (eRegulons) where master TFs are connected to a group of candidate *cis*-regulatory regions, which are further connected to a group of downstream target genes. SCENIC+ identified 74 activator eRegulons from the mouse DRG multiome dataset. The eRegulons consisted of ~14,000 regions and ~5000 genes in addition to the TFs (Supplemental Table S5). Most genes were involved in 1 or 2 regulons and controlled by <10 enhancers. Most individual enhancers were part of 1–2 eRegulons and most enhancers controlled a single gene (Supplementary Fig. S9a and b). We reasoned that for any eRegulon, the TF expression in combination with the activity of the entire enhancer module lies causally upstream of the gene expression module. To follow this logic, we visualized the eRegulon activity as a dotplot-heatmap where dot size represented the expected TF-region component of the eRegulon (the product of TF expression and accessibility score for regions in the corresponding eRegulon) with color coding the expression score of the downstream gene set of the eRegulon (Fig. 5a, Supplementary Fig. S9c). The eRegulon activity was further visualized for both gene and region sets individually together with TF expression (Supplementary Fig. S9d and e). Our results confirmed active gene regulatory networks driven by previously known key TFs, such as Runx3 for proprioceptors, Runx1 for NP1 and Zfp521 for C-LTMRs. Some eRegulons (such as Nf1, Smad1, Bclaf1, Ctcf, and Hdac8) were predicted in this analysis to be active throughout most of the neuron types, whereas others showed strong activity in a restricted set of neurons: for example, Rora among the Rxfp1, TRPM8 and proprioceptor types; Bcl11a and Bcl11b in most PEP1, PEP2, and PEP3 subtypes; Nfia in PEP2, PEP3, Aδ- and Aβ**-**types; Klf5 in the NP1, NP2, and NP3 classes; Fli1 in the NP1, NP2, and C-LTMR types; and Esrrg in the myelinated LTMRs. Overlap of target genes showed that certain eRegulons, namely Ebf4, Bcl11a, and Bcl11b; Nf1, Cbfb, and Pbx1, Nfib and Nfia; Klf6, Nfe2l1, and Klf9 shared relatively many genes among the groups. Especially the groups: Klf5, Ebf1, and Klf7 were strongly active in NP3 and Zfhx3, Ebf3, Bnc2, Bach2, Runx1, and Runx2 most strongly active in NP1 shared significant proportions of genes among combinations of them, suggesting strong cooperation among the master TFs of these regulons in defining the NP3 and NP1 neuron phenotypes (Supplementary Fig. S9f). Looking at the overlap of eRegulon enhancers, Nfia and Nfib; Bcl11a and Bcl11b; Klf5 and Klf7; and Runx1 and Runx2 used shared region sets (Supplementary Fig. S9g). Most eRegulons with low activity in the TF-region module had also low activity in the gene expression module (eg. Maff, Atf3, Klf1) (Supplementary Fig. S9c–e). Interestingly, many eRegulons had high TF-region accessibility with low gene module activity, suggesting that the eRegulon is primed but requires other factors to be actively transcribed (e.g. Rcor1, Stat1). Based on analysis of the relation between transcription factor expression, accessibility score for regions in the corresponding eRegulon, and downstream gene set of the eRegulon (Fig. 5a, b, Supplementary Fig. S9c–e) we conclude the following: (1) TF expression and accessibility is always present when there is the downstream gene set eRegulon expression. (2) However, downstream gene set expression does not always have a direct relationship to TF expression and accessibility, indicating cooperative functions of several TFs. (3) TFs can be expressed without accessibility or downstream gene expression; thus, patterns of downstream gene expression are limited by chromatin accessibility. (4) TF expression and accessibility may be present without downstream gene expression, indicating the existence of repressor TFs.

To validate the conclusions using the larger integrated atlas, we scored each of the 74 eRegulon gene modules in the atlas which verified that the gene-regulatory networks generalize beyond the multiome dataset (Fig. 5b, Supplementary Fig. S9h).

Expression of many, if not most, gene products enriched in subsets of sensory neurons and used as markers could be explained by the identified active enhancer motifs and target gene expression (Supplementary Table S6), including, for example, *Calca* (CGRP), *Ntrk1* (TrkA), *Gal*, *Npy2r*, *Chrna3*, *Scn10a*, *Th*, *Slc17a8* (Vglut3), *Cbln1*, *Agtr1a*, *Nppb*, *Osmr*, *Hrh1*, *Sstr2*, *Gfra1*, *Gfra2*, *Adcyap1*, *Bmpr1b*, *Prokr2*, *Rxfp1*, *Trpm8* and much more. Three transcriptional logics were inferred: (1) Often different transcription factors in different subtypes of neurons drive the expression of the same gene. For example, *Scn10a* was controlled by Ebf3 in Mrgprb4 neurons and NP1-NP3, while Bcl11b regulated expression in PEP1.1-PEP1.4. Similarly, expression of the mechanotransducer *Piezo2* was mainly governed by Ebf3 in pruriceptors, Onecut2 and Nfia in A-LTMRs, and Bach2 in C-LTMRs. (2) Expression of some genes was cooperatively controlled by several eRegulons, such as for example, Nfia and Nfib for *Bmpr1b* expression in PEP3 neurons and Bcl11b and Ebf4 for expression of *Sstr2* in PEP1.1 neurons. (3) Different sets of genes enriched in the neuronal subtypes (subtype gene programs) were controlled by different transcription factor eRegulons, for example in C-LTMRs; Zfp521 regulated *Th*, Bach2 regulated *Slc17a8*, Runx1 regulated *Gfra2*. Such gene programs were the most complex for PEP1.1-PEP1.4 neurons which were predicted to contain several shared eRegulons where for example Bcl11a regulated *Gal*, Bcl11b regulated *Calca*, and *Ntrk1*, Ebf4 regulated *Adcyap1* (Fig. 5a, Supplementary Fig. S9c, Supplementary Table S6). These results show that active enhancers in sensory neurons explain unique and shared molecular features and identify the transcriptional principles for maintenance of the sensory neuron heterogeneity which underlies somatosensation.

### Co-regulation gene expression networks in sensory neurons

**Fig. 6:**
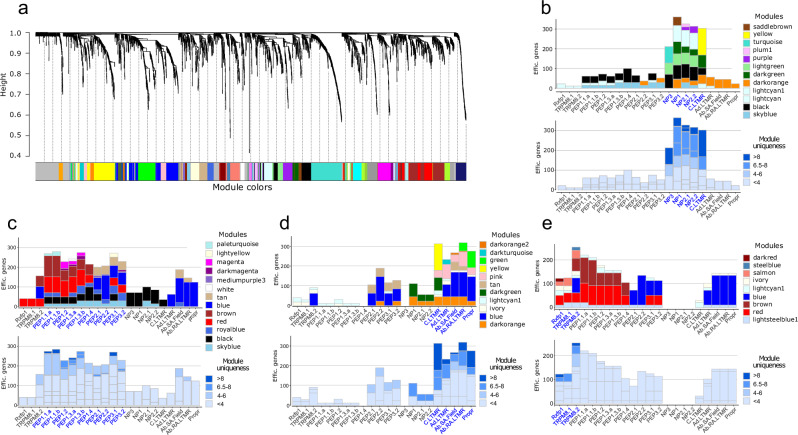
hdWGCNA gene modules and their distribution in mouse DRG neurons. **a** Dendrogram of co-expression gene network analysis with identified modules. **b**–**e** Bar plots for module representation across neuronal types. Modules present in hierarchically related neuron types are presented separately in (**b**–**e**). **b** NP + C-LTMR, **c** PEP1 + PEP2 + PEP3, **d** C-LTMR + A-LTMR+Proprioceptors, and **e** Rfxp1+Trpm8 are represented by bar plots (stacked vertically) where only active modules in given cell type group are shown. *Y*-axis is the number of genes assigned to module times module signal value (i.e. 0.5 or 1). In the upper plot, the color code represents module names, in the lower plot, bars are colored according to module uniqueness. On the *X*-axis names in blue font depict neuronal types for which all active modules are plotted. Note that C-LTMRs are plotted both with NP group **b** of clusters and other LTMRs/Proprioceptors in (**d**).

Weighted gene co-expression network analysis (WGCNA) is a method that identifies clusters of highly correlated genes (modules) across sample traits to identify biologically meaningful gene modules potentially involved in specific cellular processes. High-dimensional WGCNA (hdWGCNA)^11,36^ extends WGCNA by enabling the identification of gene co-expression modules within individual cell types across single-cell datasets by first aggregating single cells into metacells to reduce noise, then constructing co-expression networks by calculating correlation patterns between genes, clustering these patterns into modules of co-regulation network gene expression, and finally projecting module activity back to individual cells. Using hdWGCNA we identified 46 co-regulation network expression patterns (modules) (Fig. 6a). The signal from individual modules varied across clusters versus datasets (Supplementary Fig. S10a). To select modules contributing to the difference between clusters rather than datasets we analyzed individual module magnitude across clusters vs across datasets (Supplementary Fig. S10b) and found 11 that varied across datasets. These modules were excluded from further analysis. Of the remaining 35 modules one was specific for the ATF3+ neurons and another, midnightblue, represented a co-regulatory module enriched in satellite glia (Supplementary Fig. S10a). We concluded that this module arose from satellite glia contamination across neurons in the integrated atlas.

We applied a procedure assigning genes to several co-regulation networks (modules) if the correlation coefficient was similar. Out of the 1792 genes in all 46 modules, 1513 had unique assignments and 279 were assigned to between two and four modules (Supplementary Table S7). Some modules were highly specific for individual populations of neurons, while others were shared between groups of neuron types. To simplify the representation of the modular transcriptional architecture we defined module expression levels across neuron types to none, half, or full expression by assigning expression levels as 0, 0.5, or 1. Module contributions to the transcriptional identity of each neuronal type were then visualized as a series of bar plots where the height of each segment in a bar represented the product of the number of genes assigned to the given module and the module’s expression level in a given neuronal population. (Fig. 6b–e, Supplementary Fig. 10c). This analysis predicted that each neuronal population was characterized by about 250 “efficient” genes. The exceptions were the Rfxp1 and TRPM8.1 neurons whose transcriptional identity was explained by about half of this number, around 120.

The brown module was highly specific for PEP1 and its close relative, TRPM8.2. The blue module represented co-expressed genes in fast-conducting populations, including also TRPM8.2. We did not identify any pan-PEP modules; however, one module (red) was specific for Rfxp1/TRPM8 and PEP except PEP2, and another (black) was enriched in NP and PEP neurons (except PEP2.2 and PEP3.2). We found two smaller modules (41 and 29 genes, royal blue and sky blue) broadly expressed in PEP subtypes but with minor activity also in Aβ Field-LTMRs and NP2. One module, tan, was shared between all fast-conducting PEPs (PEP2/3) and Aβ**-**LTMRs. There were almost no co-expression modules shared between PEP1 and A-LTMRs and NP and Rfxp1/TRPM8 neurons. LTMR and Rfxp1/TRPM8 populations shared only one module (blue). Interestingly, mechanoreceptors with relatively low thresholds, such as C-LTMRs, NPs, and A-LTMRs, shared the dark orange module. The transcript for mechanoreceptor *Piezo2* had the second-best correlation to the summarized module expression profile as represented by one gene: the module eigengene (Supplementary Table S7, the last tab).

In scRNA-seq analyses of sensory neurons, PEP classes of neurons share many features and, on many visualizations, represent a continuum of transcriptional states. To better understand the transcriptional basis, we assessed the relative uniqueness of modules contributing to transcriptional identity. Analyzing module expression uniqueness revealed that NP neuron types and LTMRs (apart from Aβ Field-LTMRs) had greater uniqueness than any of the PEP types (Fig. 6b–e, Supplementary Fig. S10c, bar plots in shades of blue). This indicated that the transcriptional identity of neuronal populations is largely built by shared modules with a smaller contribution of modules with cell-type specificity, except for PEP neurons for which only a very small part of transcriptional identity is explained by cell type-specific modules. Thus, the identity of PEP subtypes could mostly be explained by the variability in expression levels of shared modules (Fig. 6c). These modules were often active in most PEP-type neurons and sometimes also in other populations.

We also projected the modules identified in mice to a human dataset^21^ (Supplementary Fig. S10d). Mouse modules specific to neuronal types with an obvious homolog in humans demonstrated a similar pattern across human neurons, though with lower contrast. However, modules specific for mouse neuronal types not having clear homologous neuron types in humans did not demonstrate any conserved module expression (magenta for PEP1.2, dark magenta for PEP1.2/1.3, pale turquoise for PEP1.1.b). Interestingly, the white module, highly unique for mouse type PEP1.3.a, was relatively specific in human hPEP.TRPV1/A1.1.

Gene ontology (GO) analysis of genes comprising individual modules revealed low significance (Supplementary Table S8), perhaps because of poor GO annotation related to the specific function of sensory neuron subpopulations. One of the largest modules (blue, 124 genes, present in all myelinated neuron types) had a very strong association with “peripheral” cellular component (CC) branch of terms: axon, synapse, basal dendrite, and node of Ranvier, indicating that genes of this module contributed to peripheral functions of fast conducting neurons, including genes involved in structural, electrical and synaptic functions. Apart from the blue module, only two other modules had an association with synapse-related terms: the pink module (active in LTMRs) and the green module (proprioceptors). For the other neuron classes (PEP/Rfxp1/TRPM8/NP) functions associated with the synaptic/axonal compartment were not dominated in any specific modules but shared between them.

### Epigenetic basis for co-regulated gene expression in sensory neurons

**Fig. 7:**
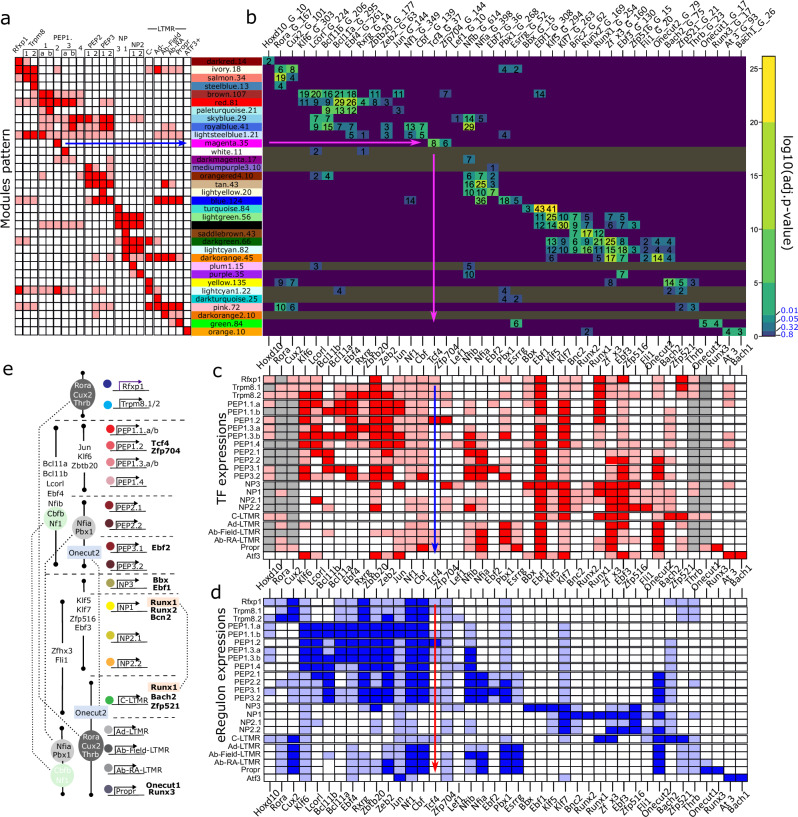
Prediction of eRegulons driving the co-regulated gene expression that generates heterogeneity of mouse DRG neurons. **a** Heatmap of modules expression profile (rows) across neuronal populations (columns). Modules names are on the right, followed by the number of genes in each module. Rows from a are projected to the right to panel (**b**). **b** Heatmap representing overlaps of gene lists comprising modules (rows) vs. eRegulons (columns). Numbers in each cell stand for a count of overlapping genes, the color of a cell is log10 of the adjusted *p*-value for overlap significance (Fischer exact test). Numbers next to the names of eRegulons above the heatmap depict the number of genes in each eRegulon. Blue numbers next to the color scalebar are adjusted *p*-value (i.e. without log10) corresponding to color change significance levels. Gray rows represent modules without significant overlapping with eRegulons (p.adj = 0.05). Columns from **b** are projected vertically down to (**c**) and (**d**). **c** Expression pattern of driver TFs for specific eRegulons across sensory clusters. Gray cells—too low signal to access TF expression level. **d** Expression pattern of all genes in the eRegulons across sensory clusters. Heatmaps **a**, **c**, and **d** represent arbitrarily digitized expression levels from no expression (white) via weak (pale color) to strong (intensive color). **e** Schematic summary of TFs control of sensory clusters based on eRegulons pattern. TFs controlling several neuronal populations not neighboring on the chart are highlighted by colored background and connected by dashed lines. TFs in bold on the right are specific for individual neuronal types.

We next examined whether the co-regulation networks were defined by the epigenetic status of the neurons. For this purpose, we intersected the co-regulation gene expression networks with target genes controlled by transcriptional motifs to see if the eRegulons can explain the co-regulated modules. We found that all except 7 WGCNA modules had at least one significant overlap with eRegulons (Fig. 7a and b) and hence, the epigenetic cell status predicted the patterns of co-regulated genes which contribute to the heterogeneity of sensory neurons. Modules (rows in Fig. 7a and b) were grouped according to the similarity of their transcriptional control, which expectedly corresponded to the profile of module expression across neuronal types. This showed, for example, that the blue module was predicted to be controlled by Nfia, Pbx1 with low significance signal from Nf1 and Esrrg; the pink was driven by Rora and Thrb with low significance contribution from Cux2 and Zfp521; the dark orange module was controlled by Zfhx3 and Onecut2 with modest contribution from Ebf3 and low confidence signal from Klf5, Runx1, Fli1 and Bach2. Thus, these predictions suggested that co-regulated gene expression patterns were largely generated by a combination of enhancer-driven GRNs, a finding in congruence with that different eRegulons can drive expression of the same gene, that several transcription factors can contribute to the expression of a specific gene and that each gene regulatory network contributes with a subset of genes defining neuronal populations. Furthermore, the same TF gene regulatory network appeared in several modules but with varying contributions, suggesting that varying levels of enhancer-driven activity contribute to heterogeneity. This was particularly evident for PEP1, PEP2, and PEP3 subtypes and the hierarchically related NP1, NP2.1, NP2.2, and NP3 subtypes.

We examined whether a restricted expression of TFs could explain enhancer-driven expression of co-regulated gene expression. For this, we examined whether the expression of the TF itself corresponded with TF enhancer-driven co-expression modules (Fig. 7c). We found that mRNA levels of the TFs largely overlapped with the cell-type specificity of corresponding co-regulated gene expression networks. However, for a few TFs, broader expressions were observed. Finally, we examined expressions in the scRNA-seq data of the predicted target genes of TF enhancers, regardless of whether they were part of a co-regulated network. This showed, with a few exceptions, a congruence of co-regulated module activity, TF expression, and expression of target genes (Fig. 7d). Most notable exceptions were Ebf1 and Klf7 where mRNAs of the TF were markedly broader expressed in most neurons while the predicted target genes (eRegulon expression), as well as co-regulated modules, had a more restricted expression, suggesting that specificity may arise from additional mechanisms. For all neurons, we identified a combinatorial code of transcriptional regulatory networks representing shared functions across different scales of the cell-type hierarchy (Fig. 7e), which combined were predicted to drive sensory neuron heterogeneity.

## Discussion

Our integrated high-quality whole-cell scRNA-seq dataset resolved misannotated subtypes of neurons, Schwann cells, and endothelial cells, and this reduced noise when examining and analyzing gene expression. Overall, the neuronal clusters corresponded to already known sensory neuron subtypes that have independently been identified in several scRNA- and snRNA-seq studies^1–5,7,16,37,38^. Furthermore, we also cleared previously unresolved molecular heterogeneity within TRPM8, PEP1.1, PEP1.3, PEP2, and PEP3 populations, each of which split into two new molecularly defined clusters. We suggest that these new clusters represent functional units, because (1) they have mutually exclusively expressed genes, (2) were confirmed in the tissue using spatial transcriptomics, (3) contain different sets of transducers and hence different predicted response profiles to stimuli, and (4) in some cases, were distinguished by marked differences in cell size. However, it should be noted that the six previously published and the new deeply sequenced “CD1” datasets that were included in the integrated atlas were established from DRGs obtained from different spinal levels and from mice with different ages. The datasets included have DRGs collected either from many or all levels (Finno, Sharma, Zeisel, Su, CD1) or from only L4 to L5 levels (Wang, Zhang) of mice with ages spanning from 4 weeks to 6 months, with the age of the Sharma dataset being unknown.

Rxfp1 neurons had the highest levels of *Trpv1* expression compared to the other neuronal types in the ganglion and the profile of these neurons corresponded to previously identified *Trpv1*^high^ neurons. *Trpv1* expression levels have been shown to be bimodal representing two separate populations of DRG neurons, a small subset expresses very high levels of *Trpv1* whereas a larger subset of DRG neurons express *Trpv1* at low levels^39^. The *Trpv1*^high^ neurons co-express *Ntrk3* and *Esr1*, consistent with the Rxfp1 neurons identified in this study. Such *TrpV1*^high^ neurons studied in the lower lumbar ganglion innervate cutaneous targets. Epidermal innervation was observed of the clitoral sheath, scrotum, prepuce, and perianal regions as well as hairy and glabrous skin of the hind limb, and was activated by innocuous heat^39^. Conditional deletion of the *Runx1* gene in nociceptors leads to a loss specifically *Trpv1* expression in the *Trpv1*^high^ neurons but is dispensable for *Trpv1*^low^ neurons^40^. We therefore concluded that Rxfp1 neurons correspond to the previously identified *Trpv1*^high^ neurons. The TRPM8.1 population of neurons corresponds to the much-studied unimodal cool sensing neurons because it expresses *Trpm8* but no other thermosensitive channel. However, measuring GCaMP signal in DRG in response to thermal stimuli of the skin has revealed a subset of Trpm8^+^ neurons that respond not only by cooling but also by elevations in skin temperature^18^. That response profile is consistent with the TRPM8.2 neurons which expressed the heat transducing channels *Trpv1*, *Trpm2*, and *Trpm3* along with *Trpm8*. These neurons could be lightly myelinated and conduct with slightly higher velocity than fully unmyelinated neurons if classified based on *Nefh* and *Cntnap1/Cntnap2* expression. Interestingly, the mouse TRPM8.2 neurons functionally correspond to a human menthol and cold-responding C-fiber type also activated by heating^41,42^. Bifurcation of the neurons expressing *Sstr2* uncovered one type with a predicted dynamic heat response (PEP1.1.a expressing *Trpv1*, *Trpa1*, *Trpm3*, and *Piezo2*) while the other is predicted to show a more extreme heat response (PEP1.1.b expressing *Trpv1*^low^ and *Trpm3*). A bimodal heat response has been reported among *Sstr2*+ neurons, some responding at 40–45 °C and others at 55 °C, although it remains unclear if neurons responding at 40 °C also respond at the higher temperature^18^. The division of PEP1.3 into PEP1.3.a and PEP1.3.b unexpectedly revealed neurons of very different sizes, with PEP1.3.a having similar mean cell diameter as A-LTMRs despite likely belonging to the C-fiber category. Mouse lines driving expression from the Chrna3 and *Adra2a* loci, both expressed in PEP1.3.a, have been used to characterize sensory neurons^17,18^. We defined two populations of neurons expressing Chrna3 (PEP1.3.a and PEP3.1). This is consistent with a previous study using a Chrna3-GFP reporter^17^ which finds *Chrna3*^high^ and *Chrna3*^low^ neurons with summation of two Gaussian distributions. *Chrna3* is highly expressed in PEP1.3.a and lowly expressed in PEP3.1 consistent with high and low expressing neurons representing different sensory neuron types. The *Chrna3*^high^ neurons include C-fiber silent nociceptors innervating muscle, colon, urinary bladder, and knee joint, but not skin^17^. However, we did not find that PEP1.3.a expresses *Piezo2*, as reported for the *Chrna3*^high^ neurons, a discrepancy that could reflect differences in *Piezo2* expression along the spinal levels: Prato et al^17^ studied sacral sensory ganglia which are likely underrepresented in the individual scRNA studies used to generate the integrated atlas. However, this discrepancy may also relate to the fact that the Chrna3-GFP is a transgenic mouse line that might not fully recapitulate actual *Chrna3* expression. The PEP1.3.a neurons also expressed *Adra2a*. Robust labeling in internal organs, including bladder and colon, but not of limb or trunk skin, was observed using an *Adra2a*^*T2a-CreER*^ reporter mouse strain, consistent with *Chrna3*^high^ neurons^18^.

PEP2.1 and PEP2.2 populations both expressed markers for myelinated axons, aligned with previous predictions of the conflated PEP2 neurons. Both types expressed *Piezo2* with PEP2.1 also expressing low levels of *Trpv1* but no other thermosensitive TRP channels. DRG neurons marked in *Smr2*^*T2a-*Cre^ reporter mice innervate skin, contain large arborizations, have high thresholds to mechanical stimulus, and respond to heat and hence are Aδ-HTMR/Heat neurons^18^*. Smr2* is expressed in PEP2.1 but not PEP2.2, consistent with *Trpv1* expression in PEP2.1 neurons. The lack of any thermosensitive TRP channels in PEP2.2 suggests that this population represents an Aδ-HTMR without responsiveness to thermal stimulation. Intriguingly, the closest hierarchically related neuron population is the Aβ Field-LTMRs, yet PEP2.2 expressed numerous genes associated with nociceptors including, for example, *Prdm12*, *Scn10a* and *Ntrk1*. Recently, the use of intersectional genetics to study *Ntrk3*^*+*^ sensory neurons expressing *Scn10a* revealed these to be myelinated high-threshold mechanoreceptors that terminate peripherally as free nerve endings and circumferential endings in hair follicles with central termination of inner lamina II, laminae III and IV, thus partially overlapping with A-LTMRs^43^. Activation of these neurons induced the nociceptive withdrawal reflex without aversive behavior. Beyond the expression of *Ntrk3*, these neurons expressed *Nefh* and were negative for *Trpv1* which aligns with PEP2.2 neurons.

*Bmpr1b* and *Kit* expressing neurons terminate as circumferential endings on hair follicles, are Aδ-HTMRs, and respond to hair pull^6,18,44^. *Bmpr1b* and *Kit* were expressed in both PEP3.1 and PEP3.2 neurons, the former likely terminating as circumferential endings conveying responses to hair pull while the latter expressed markers consistent with a bias towards innervation of internal organs and deep somatic tissues, given that *Prokr2* is a marker of both unmyelinated and myelinated afferents innervating deep tissues and organs^19,45,46^.

The current study, nor any other study, has identified molecular heterogeneity within the two subtypes of Aβ-LTMRs, i.e. the Aβ SA-LTMRs and Aβ RA-LTMRs. Yet it is known that Aβ SA-LTMRs can terminate as the Merkel cell neurite complexes detecting forces and fine form or as circumferential endings (called field-receptors) also encoding skin touch while Aβ RA-LTMRs can terminate as lanceolate endings, or within Meissner and Pacinian corpuscles. Thus, a single transcriptionally defined group of Aβ-SA LTMRs seems to innervate both Merkel cells and form circumferential endings of hair follicles, and similarly, a single transcription type appears to terminate as lanceolate endings, in Meissner or Pacinian corpuscles. The failure of finding transcriptional types corresponding to the different nerve terminal morphology and physiology of these LTMRs could either be due to a lack of power for resolving heterogeneity or an absence of molecular heterogeneity, in which case the morphology of nerve terminals and physiology could be determined locally within the target tissue.

Quantification of cell soma size using MERSCOPE allowed direct comparative data on the relation of specific neuronal types among all neurons in the DRG. We found that Aβ Field-LTMR neurons had the largest cell soma while the remaining A-LTMRs, proprioceptors, A-fiber HTMRs, both PEP2 types, PEP3.2, and unexpectedly the C-fiber PEP1.3 and PEP1.4 also were large size neurons. The variation in size is thought to correlate with the degree of axon myelination, thereby influencing the conduction velocity. However, our results indicated a disconnect between soma size and myelination. We also observed this disconnect among the smallest neurons where TRPM8.2 neurons could be lightly myelinated (*Nefh*, *Thy1*, and *Cntnap1/Cntnap2* expression was similar to PEP2, PEP3, and A-LTMRs and proprioceptors).

We found that the main spatial organizational feature of neurons in relation to other neurons in the DRG is that neurons of the same and similar types are closest to each other. Neurons generated from a single neural crest cell are often clustered within the chick DRG, suggesting that many neurons arise by cell division after migration is nearly complete^15^. Also in the mouse, progenitor cells fated to the sensory neuron lineage continue dividing after condensation of the DRG and after neural crest migration is complete^14^. Thus, we believe that the clustering of the same type of sensory neurons is a result of the formation of the DRG during development. We also found that neuronal subtypes were organized in zones where Aδ-LTMR, Aβ Field, Aβ RA-LTMR, proprioceptors, PEP2 and PEP3 neurons were closest to the outer perimeter of the ganglion while the TRPM8 types and NP3 were embedded deepest within the ganglion and the other neuronal types in a zone in between. Inter-neuronal ephaptic coupling or cross-excitation between DRG neurons possibly mediated by satellite glia has been proposed^47–49^, in which case the zonation and spatial clustering of similar neuronal types could lead to a functional impact. Quantification of the abundance of different sensory neuron populations has been hampered by the inability to study all neuronal types at the same time. Using MERSCOPE, we classified all mouse neurons at the same time in the tissue. Our results show that there is a nearly 30-fold difference in proportion between the most and the least abundant types (NP1 and PEP1.2, respectively), and three neuronal types, NP1, C-LTMR, NP3, together represent more than half of all neurons in the DRG. C-fiber neurons involved in interoception and nociception, such as the PEP1, PEP2, and PEP3 subtypes, and thermoreceptor neurons such as the TRPM8 and Rxfp1 were comparably rare, each representing just a few percent of the ganglionic neurons.

DRG does not only contain cell bodies of the primary sensory neuron but also a variety of other cell types such as glia (satellite cells, myelinating, non-myelinating), endothelial cells, pericytes, and smooth muscle cells of the arterioles and capillaries supplying the DRG. In addition, there are fibroblasts forming connective tissue around nerves, bundles of nerves, and meningeal tissue around the ganglion as well as immune cells^50^. These cell types were identified, their relative abundance quantified, and spatial organization determined. The identification of SMA^+^ cells around large vessels is consistent with ganglionic arterioles supporting the capillary bed^51^, identified by the presence of arteriolar endothelial cells and pericytes. The concept of “metabolic zonation,” as defined by positional proximity of cells to perfused blood vessels can shape cell metabolism and function^52^. We analyzed whether sensory neurons might display different metabolic “niches” and vulnerabilities depending on their respective localization. We were unable to identify co-regulated gene modules or enhancer-driven gene expression corresponding to this relationship, such as oxygenation and cellular processes of metabolic energetics and potential availability of key metabolites. However, metabolic zonation cannot be excluded in situations of challenge, such as compression, injury, and/or inflammation.

Analyzing chromatin accessibility in neurons revealed that accessible regions close to genes reliably predicted downstream gene expression and when instead selecting and analyzing top markers from the integrated atlas, accessibility, and gene expression reliably overlapped. This suggests high quality in our snATAC-seq dataset. Inferring variability of transcription factor-associated accessibility across the neurons using chromVAR revealed complex patterns of TF families within different classes of neurons. However, it was difficult to assess their importance. We therefore analyzed eRegulon activity evaluating accessibility, transcription factor expression as well as downstream gene expression using SCENIC + . By this, we were able to identify transcription factor binding sites and judge whether the accessible sites were active or inactive based on the regulons controlled by each transcription factor. The most overt observation were patterns each containing several transcription factors enriched in related neuronal types and present with varying activities. These blocks of eRegulons were present at different resolutions across the hierarchical relationship. This indicates that the maintenance of diversity is driven by a combination of blocks of eRegulons and within-block variations of activity. For example, PEP2/3 neurons contained a block of eRegulons shared with all neurons except NP1-3, another shared with PEP1 neurons, as well as a PEP2/3 block shared with other myelinated neurons and some cell-type specific eRegulons. We also identified several transcription factors enriched in one or a few neuronal subtypes, including for example previously studied Zfp521^53^, Cux2^53^, Onecut1, Onecut2, Runx1^53^, Runx3. Interestingly, key TFs predicted to drive segregation and diversification in human DRG neurons during embryonic development (gestation weeks 7–21)^54^ were also found to drive eRegulon activity in adult mouse DRG neurons (Rora in TRPM8 neurons, Nfia/Nfiab in PEP2, PEP3 neurons, Klf5 and Zfx3 in pruriceptors, Bach2 instead of BACH1 in C-LTMRs). However, we found numerous other transcription factors regulating key genes defining cell type marker gene, expression of transducers (e.g. regulation of *Piezo2* by Ebf3+Zfh3 in NP types, Onecut2+Nfia in PEP2/3, Lcorl+Ebf4 in PEP1 types, *Trpm8* by Rora in TRPM8/Rxfp1, *Trpv1* by Ebf3 in NP1/2, *Trpm2* by Bbx in NP3, *Trpm3* by Zbfb20, Asic1/Asic2/Asic3 by different TFs in different cells), axon caliber and myelination (e.g. regulation of *Nefh*, *Thy1*, *Cntnap1* by Nfia in all myelinated neurons) and other genes known to contribute to neuronal hyperexcitability, sensitization, and chronic pain in nociceptors (e.g. regulation of *Cacna2d1* by Bcl11a in PEP1, *Ntrk1* by Lcorl+Bcl11b in PEP1-3 and Nfib in PEP2/3). Thus, individual key genes defining the response profile and function can often be regulated by a shared mechanism in related neuronal subtypes and yet part of a different GRN in another cell type. Because of this, tractable approaches targeting transcription factors for clinical use^55^ could open new analgesic strategies selectively affecting gene expression underlying hyperexcitability and pain in causative neurons.

Several eRegulons displayed accessible chromatin and transcription factor expression, yet did not show expression activity in the downstream-regulated genes. Such included for example STAT1, Rcorl, Jun, Nfe2l1. Interestingly, these are part of pathways activated by signaling: STAT1 by viral infections inducing the cellular anti-viral defense, Rcor1 as part of a complex recruited by REST following injury in primary sensory neurons that may contribute to hyperexcitability^56^, Fosl2/Jun activation by an axonal injury that promotes axonal regeneration^57^ and Nfe2l1 that translocate to nucleus upon cellular stress and induces genes necessary for stress adaptation^58^. Therefore, we interpret this accessible chromatin without target gene expression as primed to respond to environmental challenges. These primed genes rarely showed any cell-type-specific patterns of accessibility, indicating similar responses across all sensory neurons upon challenge.

The identification of co-regulated modules using WGCNA revealed the existence of modules organized hierarchically across molecularly related populations of neurons as well as across types with shared properties, such as for example myelinated neurons regardless of whether nociceptors or LTMRs. Integration of co-regulated gene modules with snATAC-seq data revealed that eRegulons with shared activity patterns in the neurons work together to drive the different co-regulated patterns of expression. Often, but not always, were transcription factor expressions limited to the neurons with target gene expressions. Discrepancies can be caused by the cooperation of more than one transcription factor and/or repressor activity constraining target gene expression. Nevertheless, our data predicts that cell type heterogeneity is generated by the regulation of a unique combination of co-regulated modules, each driven by one or more transcription factors. Most are shared by other neuronal types, and a few are cell-type unique. Thus, this analysis suggests the logic for how sensory neuron heterogeneity is transcriptionally created; that heterogeneity is generated by the existence of layered co-expressed gene modules, most shared but with varying contributions across different scales of neuronal types, with cell-type specific co-expression relationship as an exception.

## Methods

### Animals

Wild-type female and male C57BL/6JRj mice aged 8–10 weeks were obtained from Janiver-Labs and were kept under standard conditions on a 12/12-h light–dark cycle with free access to food and water. The experiments were conducted in accordance with Swedish policy for the use of research animals and were approved by a local ethical committee (Stockholms Norra djurföröksetiska nämnd). We have complied with all relevant ethical regulations for animal use. A total of 14 animals were used to generate the original data in this study. Sample sizes were not statistically predetermined; instead, they were selected based on previous knowledge from similar studies conducted by the authors.

### Nuclei isolation

12 mice (*n* = 6 males, *n* = 6 females, 8–10 weeks) were randomly divided into four biological replicates. No further confounders were controlled as there were no treatment and control groups in the study. Frozen DRG from the mice were pooled and thawn on wet ice in 300 µl homogenization buffer (10 mM Tris pH 8.0, 250 mM sucrose, 25 mM KCl, 5 mM MgCl_2_, 0.1 mM DTT, 0.1% Triton-X-100, 0.2 U/µl RNasin RNase inhibitor (Promega), 1x Protease inhibitor cocktail (Promega)). The tissue was motor ground (20× 3 s) and incubated on ice for 10 min followed by 20× douncing with pestle A and 20× pestle B and another 5 min incubation on ice. Cells were then filtered through a 100, 50 µm and finally, 30 µm cell strainer (BD falcon), and homogenization buffer was added to a final volume of 10 ml before centrifugation at 1500 × *g*, 10 min, 4 °C. For debris removal a 25–29% iodixanol (in 60 mM Tris pH 8.0, 250 mM sucrose, 150 mM KCl, 30 mM MgCl_2_) gradient centrifugation was performed for 25 min at 13,500 × *g*, 4 °C. The pellet was resuspended in blocking buffer I (1x PBS pH 7.4, 1% BSA, 0.2 U/µl RNase inhibitor) and stained with anti-NeuN-PE antibody (Millipore, FCMAB317PE) at 1:250 dilution for 30 min on ice. Afterward, the sample was centrifuged at 500 × *g*, 4 °C for 5 min and resuspended in clean blocking buffer II (1X PBS pH 7.4, 2.5% BSA, 0.2 U/µl RNase inhibitor) and DAPI was added to a final concentration of 0.1 µg/ ml. NeuN + /DAPI+ nuclei were sorted using fluorescence-activated cell sorting (FACS, BD FACS Aria Fusion), centrifuged (500 × *g*, 5 min, 4 °C), and resuspended in 1X diluted nuclei buffer (10X Genomics) and counted.

### Single-nuclei multi-omics sequencing

Multi-omics sequencing was performed using the 10X Genomics Chromium Next GEM Single Cell Multiome ATAC + Gene Expression Kit. In short, a targeted number of sorted nuclei (1000–5000) was treated with Tn5 transposase for 60 min at 37 °C for DNA fragmentation and insertion of adaptor sequences into open chromatin of the nuclei. Nuclei suspension was loaded onto Chromium Next GEM Chip J for single nuclei droplet generation using Chromium Controller. Reverse transcription, cDNA amplification, Gene expression, and ATAC library construction were performed according to the user guide provided by the manufacturer. Pooled ATAC libraries were sequenced on Illumina sequencing platform NovaSeq 6000 system on one S1-100 (v1.5) flowcell with a custom read set-up of 50-8-24-49 bp and pooled gene expression libraries were sequenced on Illumina sequencing platform NovaSeq 6000 system on one lane of a S4-300 (v1.5) flowcell with a custom read set-up of 28-10-10-90 bp in National Genomics Infrastructure (SciLifeLab). Raw sequencing data were de-multiplexed, converted into fastq format, and aligned to mouse reference mm10 using the CellRanger ARC (v.2.0) to generate gene-cell and fragment-cell matrices.

### Single-nuclei multi-omics analysis

Gene expression and ATAC data matrices from CellRanger-ARC output were imported into R (v4.4) and analyzed primarily using Seurat (v5.1) (https://github.com/satijalab/seurat) and Signac (v.1.13) (https://github.com/stuart-lab/signac). The nuclei were first processed on the RNA side following a standard Seurat pipeline of SCTransform; RunPCA; RunUMAP; FindNeighbors; FindClusters (dims = 1:20, resolution = 0.2). All nuclei with <1000 UMI or >3% of reads originating from mitochondrial genes were filtered out. To assign the nuclei to the defined neuron types, we trained a classifier using the integrated atlas scRNA-seq data with scPred (v.1.9.2) (https://github.com/powellgenomicslab/scPred) using mixture discriminant analysis (MDA) as the underlying model. To have a homogeneous dataset for the model generation, we used only the data originating from the Sharma et al. study^2^. After assigning labels with the model, all neurons that failed to reach the cut-off assignment score (0.55) were filtered out. We then called peaks on the ATAC data using MACS2 individually for each cell type. After this, the data was further filtered to only include nuclei with >1000 peaks, >10% fragments in peaks, and Ta SS enrichment score >4. Peak annotation was done using Homer (annotatePeaks.pl). The filtered data was clustered in the ATAC modality using term frequency-inverse document frequency (TF-IDF) followed by singular value decomposition (SVD) using the most frequently observed features (RunTFIDF, FindTopFeatures, RunSVD) followed by creating the UMAP (RunUMAP). In addition, we ran a weighted nearest neighbor (WNN) analysis learning the cell-specific weights from the individual modalities and constructing a WNN graph that integrates the RNA and ATAC modalities. For this, we used Seurat::FindMultiModalNeighbors(reduction.list = list(“pca”, “lsi”), dims.list = list(1:20, 2:20)) followed by Seurat::RunUMAP. Gene activity (chromatin accessibility in the gene body and promoter) was calculated using Signac::GeneActivity. Marker genes for gene activity were defined using Seurat::FindAllMarkers and the top markers from this list were extracted based on adjusted *p*-value ignoring any genes starting with “Gm” or “AC”. Cell-type markers from the integrated atlas were extracted in a similar manner but for RNA. To visualize the gene activity and gene expression markers in the multiome dataset as heatmaps, the data were aggregated on cell type level in both “Activity” and “RNA” assays using Seurat:: AggregateExpression. Chromvar (https://github.com/GreenleafLab/chromVAR) was run using the Seurat wrapper function RunChromvar using motifs from chromVARmotifs::mouse_pwms_v2 and added as an assay to the Seurat object. Differential activity for motifs was then calculated using Seurat::FindAllMarkers with mean.fxn = rowMeans. For each motif, the transcription factor and family were assigned from the CIS-BP Database (http://cisbp2.ccbr.utoronto.ca/matchlist.php) and the mean deviation was calculated for each transcription family. The transcription family level deviations were used as the final output. eRegulon analysis using SCENIC+ (https://github.com/aertslab/scenicplus) was performed as outlined on the software home page (https://scenicplus.readthedocs.io/en/latest/index.html) using the precomputed cisTarget databases for mouse. The resulting eRegulons were filtered to only include regulons with an extended annotation if there is no direct annotation available and regulons for which the region-to-gene correlation is positive. The resulting eRegulon activity matrices (region and gene-based) were included as assays in the Seurat object. Modules scores for the eRegulon gene expression activities were calculated for each nucleus in the data using the Seurat::AddModuleScore function. Similar scores for the gene-based eRegulons were calculated for all cells in the integrated atlas. To visualize the activity of each eRegulon in the multiome data (Fig. 5a, Supplementary Fig. S9c), we used a dot plot-heatmap where the dot size represents the product of the mean TF expression scaled between neuron types (scaled to 0–1) and the eRegulon region module score (scaled to 0–1) for that TF. The color of each dot then represents the scaled gene expression activity score of the combined downstream genes of the regulon. We also plotted the data as dot plot-heatmaps where dot size is the scaled TF expression (0–1, between neuron types) and color is either eRegulon gene set or region set activity (Supplementary Fig. S8d and e), A similar dot plot-heatmap with combined TF-region score as size and eRegulon gene set activity score as color was created for the integrated atlas (Supplementary Fig S9h).

### MERSCOPE spatial transcriptomics sample preparation and imaging

Wild-type mice (*n* = 2 females, 8 weeks) were sacrificed with an overdose of isoflurane and cervical dislocation and DRGs were prepared on ice and mounted in optimal cutting temperature cryomount (HistoLab AB). For spatial transcriptomic, the Vizgen user guide protocol for non-resistant tissue was followed. The tissue was sectioned with a cryostat (NX70, Thermo Fisher Scientific) at a thickness of 10 µm before mounting on a MERSCOPE slide (Vizgen) and short 5 min incubation at −20 °C. Afterward, the tissue was fixed in 5 mL 4% PFA for 15 min at room temperature and washed three times in 1X PBS for 5 min each. The tissue was then permeabilized in 5 mL 70% Ethanol overnight at 4 °C following a 2 min wash in sample prep wash buffer (Vizgen) and 30 min incubation in formamide buffer (Vizgen) at 37 °C. For probe hybridization 50 µl of a custom 300 gene probe panel (Supplementary Table S2) was added to the section, covered with parafilm, and incubated at 37 °C for 48 h. Sections were washed twice in formamide buffer at 47 °C for 30 min and once in sample prep wash buffer for 2 min at room temperature. The tissue section was then covered in 4.9 mL gel embedding solution (5 mL gel embedding premix (Vizgen), 25 µl 10% APS, 2.5 µl TEMED) for 1 min. Solution was discarded and 50 µl Gel embedding solution was added to the section and covered with a GelSlick coated coverslip followed by a 90 min incubation at room temperature. Afterward, the coverslip was removed and the tissue was cleared in clearing solution (5 mL clearing premix (Vizgen), 50 µl Proteinase K (Sigma)) at 37 °C for 24 h. Before imaging, the section was washed twice with sample prep wash buffer and stained with DAPI and PolyT staining reagent (Vizgen) for 15 min at room temperature, washed in formamide wash buffer for 10 min, sample prep wash buffer for 5 min and immediately proceeded for imaging in the Vizgen MERSCOPE instrument following the MERSCOPE Instrument User Guide (91600001 Rev G) and using the MERSCOPE 300 gene imaging kit (10400005). MERSCOPE Visualizer, Zen Black 2010/ Zen Blue v2.3 and v3.1 (Zeiss), and LAS X (Leica) software suites were used in the image analyses.

### Segmentation of MERSCOPE data

For segmentation of the MERFISH data, the Vizgen Post Processing tool (VPT) and Cellpose2^22^ (https://github.com/MouseLand/cellpose) were used, following a Vizgen segmentation guide https://vizgen.github.io/vizgen-postprocessing/analysis_vignettes/segmentation_heart_dataset_cellpose2.html) in Python 3.11. In short, 15 images of DRG sections were exported and semi-manually segmented in Cellpose2. A custom model was trained based on the manual segmentation and the full dataset was re-segmented with the following settings: nuclear_channel = DAPI, entity_fill_channel = PolyT, cell diameter (pixels) = 102, flow_threshold = 0.8, cellprob_theshold = −5.5, stitch_threshold = 0.0. The newly generated metadata files were used to update the vzg file, uploaded in the Vizgen Visualizer software, and used for visualization of the spatial transcriptomics data and exporting example images.

### MERSCOPE data clustering analysis

A Seurat object was created from the expression matrix of the MERFISH spatial transcriptomics data. The data was then processed using the default Seurat scRNA-seq pipeline using SCTransform, dims = 1:20 and resolution = 0.5. To identify putative cell types in the data, we used a supervised learning approach, like the one used previously for multiome data. We first combined the Sharma et al. subset of the integrated atlas with the non-neuronal control scRNA-seq data from Su et al.^16^. We then created a classifier for this data and used it to learn cell type labels for the MERFISH data. Because the MERFISH data contained only a total of 300 genes, we relaxed the cut-off score for assignment and first assigned each cell to the closest scoring cell type. We then manually checked the matching and adjusted labels where it was necessary. After this, we removed all cells that matched poorly to any cell type even if labeled by the relaxed assignment. We then split the data into neuronal and nonneuronal parts and repeated the clustering procedures for each to obtain UMAP projections but keeping the learned cell type labels. Marker genes for both neuronal and nonneuronal cell types were extracted using Seurat::FindAllMarkers and plotted as dot plots.

#### MERSCOPE spatial analysis

To quantify the spatial proximity of different cell types for FISH-based spatial transcriptomics, we updated the package of scCAMEL-VICUNA (Visualization of Inter-Cellular Untangling and Annotation).

### scCAMEL-VICUNA for measuring pairwise distance among cell types

To examine the spatial organization and proximity between different cell types within the tissue, we calculated the pairwise distances between the centroids of each cell. This analysis was designed to quantify the spatial relationships between cell types and their spatial distribution patterns. We first obtained a spatial data set containing the centroid coordinates (*x*, y) of each individual cell in the tissue. Each cell was labeled with a corresponding cell type. In addition, the volume of each cell was included in the data set to account for possible size-related fluctuations during distance normalization. The data set was structured as a dataframe, with each row representing a single cell and the columns indicating the *X* and *Y* coordinates, cell type, and volume of the cell. To calculate the spatial distances between cell types, we first calculated the pairwise Euclidean distance between the centroids of each cell. The Euclidean distance between two cells, *i* and j, was calculated using the following formula:

$${{{\rm{d}}}}{{\_}}{{{\rm{ij}}}}={{{\rm{s}}}}{{{\rm{q}}}}{{{\rm{r}}}}{{{\rm{t}}}}(({{{\rm{x}}}}\_{{{\rm{i}}}}-{{{\rm{x}}}}\_{{{\rm{j}}}}){^\wedge} 2+({{{\rm{y}}}}\_{{{\rm{i}}}}-{{{\rm{y}}}}\_{{{\rm{j}}}}){^\wedge} 2)$$

where x_i and x_j represent the x coordinates of cells i and j, and y_i and y_j represents their y coordinates. This step was performed for all possible cell pairs to create a comprehensive distance matrix representing the spatial proximity of each cell to every other cell in the data set. Since the cells can be of different sizes, we normalized the calculated distances. For each pair of cells *i* and *j*, we calculated the normalized distance as follows:

$${{{\rm{d}}}}{{\_}}{{{\rm{ij}}}},{{{\rm{norm}}}}={{{\rm{d}}}}{{\_}}{{{\rm{ij}}}}/{{{\rm{avg}}}}{{\_}}{{{\rm{volume}}}}{{\_}}{{{\rm{ij}}}}$$

where avg_volume_ij is the average of the volumes of the two cells, calculated as

$${{{\rm{avg}}}}{{\_}}{{{\rm{volume}}}}{{\_}}{{{\rm{ij}}}}=\left({{{\rm{volume}}}}{{\_}}{{{\rm{i}}}}+{{{\rm{volume}}}}{{\_}}{{{\rm{j}}}}\right)/2$$

The pairwise normalized distances were stored in a distance matrix, where each row and each column represented a single cell and each entry in the matrix corresponded to the normalized Euclidean distance between the two cells. This matrix provided a comprehensive overview of the spatial relationships between all cells in the tissue. Since the distance between a cell and itself is zero, the diagonal of the matrix was filled with zeros. To obtain the average spatial distances between different cell types, we grouped the cells according to their type and calculated the average distance between cells of different types. Specifically, for each pair of cell types (e.g., type A and type B), we calculated the mean of all pairwise distances between type A cells and type B cells. These average distances were organized in a table from cell type to cell type. Cell type distance matrix. The Euclidean distance between two points is symmetric (d_ij = d_ji). The final cell type distance matrix was used for further analysis, such as identifying spatial clusters of cell types that tend to be closer together and visualizing spatial relationships. The matrix was also used in downstream analyses such as hierarchical clustering and correlation analysis to further investigate spatial organization patterns within the tissue.

### scCAMEL-VICUNA for measuring distance from each cell type to the tissue edge

To understand the spatial distribution of cell types, we quantified the distance of each cell to the tissue edge. This analysis revealed whether certain cell types tend to localize near the boundaries or are evenly distributed. The tissue boundary was defined as a two-dimensional polygonal shape created from image data or spatial annotations. The ConvexHull algorithm was used to build the tissue edge, enclosing all cell positions with the smallest convex polygon. Each cell was represented by its centroid (*x*, *y*), which was derived from the spatial dataset. The data set was structured as a dataframe, with each row containing the cell’s coordinates and cell type, allowing clear identification of the location of each cell. We calculated the shortest distance from each cell centroid to the edge of the tissue using geometric methods. For each cell, the minimum Euclidean distance to the polygon boundary was calculated: 1. The Euclidean distance was calculated to each segment of the boundary; 2. The shortest distance was taken as the distance to the edge of the tissue. The Shapely package was used for these geometric operations. The boundary was represented as a polygon and each cell centroid as a point. The distance method efficiently calculated the shortest distance from each centroid to the boundary. The calculated distances were aggregated by cell type. For each cell type, the average and median distances were calculated to identify trends, such as whether certain cell types were closer to the border or deeper in the tissue. The aggregated data was visualized to reveal spatial trends.

### Single-cell RNA-seq (10x Genomics) data curation and analysis

scRNA-seq datasets of mouse DRG neurons generated using the 10x Genomics Chromium 3’ were searched and downloaded from GEO (October 2022). Each dataset was processed to a Seurat object and checked for possible remaining low-quality cells. Any cells with more than 20% of transcripts originating from mt-DNA or less than 2000 detected genes, were removed. Furthermore, we removed cells with high expression of the gene “Apoe”, indicating contamination with satellite glial cells in DRG neurons. We also removed cells with low expression of the neuronal marker “Rbfox3”. Any samples originating from disruptive treatment conditions were removed.

### Integrated atlas

For integrated DRG atlas, we used five published datasets, which we refer to as “Finno”^1^, “Sharma”^2^, “Wang”^3^, “Zeisel”^4^, “Zhang”^5^, and two that originated from our laboratory which we refer to as “CD1” and “Su”^16^. First, we co-clustered all datasets using Seurat (v 4.3.0), using the R-package harmony (v0.1) (https://github.com/immunogenomics/harmony) for integration. Each dataset had pre-assigned clusters from corresponding publications. We used these annotations to transfer labels between all datasets. For this, we created classifiers trained with each individual dataset using scPred (v1.92). In other words, each cell had its original assignment (from the dataset of the origin) and then got a transferred cluster assignment from each of the other datasets. Correspondence between clusters from different datasets was obvious though different nomenclatures. This gave us over 49,000 cells in the integrated dataset. To further clean input datasets we kept only those cells that were assigned to homological clusters according to all individual datasets. This criterion was used for all clusters except A-LTMRs since assignment in different datasets to this group of clusters was not reliable. 45,382 cells passed this filtering step and were used for the final integration (2836 most variable genes shared between all datasets, harmony procedure, n.pc = 35). After cluster detection (resolution 3.4) followed by standard procedures of cluster fusion (re-fusing clusters when no reasonable separation between neighboring clusters could not be observed based on markers and when clusters were dominated by one dataset) we obtained 29 clusters. Four small clusters (52, 55, 58, and 62) were kept in the final Seurat object but excluded from follow-up analysis: these were very small and compact clusters within known neuronal clusters but represented by cells from several datasets. Out of the other 25 clusters, there were 23 neuronal (in total 44,274 cells, including the ATF3+ cluster), Schwann cells (379), and endothelial cells (218) clusters. The Schwann (glial) cell and endothelial cell clusters represented most probably neurons strongly contaminated by signal from corresponding non-neuronal cells (since they were assigned as neurons in the input datasets and passed through our filtering procedure). Dendrogram for the DRG neuron cell types was built based on the 50 principal components used in the clustering. Distances between cell types were calculated using the R function stats::dist with method = “euclidian”. Hierarchical clustering was done using the R function stats::hclust with method = “complete”. The final dendrogram was built using the R function ape::as.phylo with type = “fan”. The integrated atlas marker dot plot was created as follows. First, the atlas was downsampled to the smallest size cell type in numbers (349, Rxfp1) for each cell type. Positive gene markers for each cell type were then calculated using Seurat::FindAllMarkers function using the Wilcoxon rank sum test with |log2FC| > 0.25 and adjusted *p*-value < 0.05 as cut-offs. The markers were ordered in an ascending direction based on adjusted *p*-value and the first five markers for each cell type were plotted using Seurat::DotPlot. The sex of each individual cell in the integrated atlas was predicted using the R-package CellXY (https://github.com/phipsonlab/cellXY) following the authors’ vignette. The proportional differences between male and female cells within each celltype were analyzed using scCODA (https://github.com/theislab/scCODA).

### Cross-species cell type similarity estimation using probabilistic scoring

The analysis was conducted using the scCAMEL toolkit, a neural network-based single-cell analysis package, available at: https://sccamel.readthedocs.io/. The calculation followed previously described methods^21,59^. Briefly, a vanilla neural network model was developed for cell type classification. During model training, cell-cycle-related genes were excluded, and the most variable features were computed. Additionally, marker genes for each cell type were ranked based on two heuristics: fold change and enrichment score change, both reflecting cell type specificity. The ranked marker genes and the most variable genes were combined, log-transformed, and scaled using min–max normalization before training. The architecture of the neural network model and its parameters are detailed in the SWAPLINE package. Model accuracy was assessed by monitoring the learning curve across epochs and using *k*-fold cross-validation (*k* = 3). The optimal learning rate and the number of epochs were selected at the point where the learning curve reached an accuracy plateau. For the integration task, we first selected one dataset from our dataset pool and trained a neural network classifier to learn the transcriptional features for each cell type in that dataset. Using this trained model, we calculated probabilistic scores for all cell types. Next, we treated every other dataset in the pool as a query, applying the trained classifier to compute probabilistic scores for each cell in those datasets. We then repeated this process, each time choosing a different dataset from the pool as the training reference, until every dataset had served as a training reference at least once. After deriving probabilistic scores for all cells across all datasets, we combined these scores and performed principal component analysis (PCA). Using the elbow method, we selected the top 15 principal components to define our latent space. We then identified the top 100 nearest neighbors based on these principal components and constructed the correlation metric between cell-type pairs. For visualization, we applied a power transformation (power = 1.25) and *Z*-score scaling to the columns of the heatmap.

### hdWGCNA

Integrated atlas was used as an input for hdWGCNA (v0.2.23) (https://smorabit.github.io/hdWGCNA/). Metacells were generated from 24 neuronal clusters (including ATF3+) with following parameters: MetacellsByGroups(*k* = 50, max_shared = 15, min_cells = 261, target_metacells = 40, reduction = “harmony”, assay = ‘RNA’). Network construction was performed with following parameters: ConstructNetwork(soft_power = 16, deepSplit = 4, detectCutHeight = 0.998, minModuleSize = 12, consensusQuantile = 0.3, cutHeight = 0.2, pamStage = T). Module connectivity was run as ModuleConnectivity(corFnc = ‘cor’, corOptions = “use = ‘p’“). On obtaining the kME table we used our own redundant procedure for assigning genes to modules: each gene we assigned first to a module to which it demonstrated the highest correlation coefficient, *R*_max_. Then the gene was assigned to all modules to which it had a correlation coefficient above 0.95*R_max_. The threshold for assigning genes to any module was 0.3. To quantify if a module contributes more to differentiating clusters versus datasets we determined module magnitude in the following way: mean and standard deviation for each module eigengene were calculated for each cluster and dataset. The magnitude for a module was calculated as the difference between the eigengene means value for clusters with the highest and lowest value (the same for datasets). Then these values were divided by the mean of standard deviations across all clusters (or datasets) for correspondent module eigengene. This value is referred to as the “relative magnitude” of a module across clusters or datasets and it was used for plotting on Supplementary Fig. S10b and filtering modules by contributing to cluster difference versus datasets (the threshold value is 3.7 for relative magnitude across clusters). For modules bar plot (Fig. 6b-e, Supplementary Fig. S10c) we “digitalized” modules eigengene signal across clusters. 0 was assigned if the mean of the eigengene value for the given cluster was below 33% of the module magnitude, 0.5—if the mean was within interval 33–67% and 1 if it was above 67% of the module magnitude. To analyze how mouse modules are projected to human data^21^, we used Seurat’s function AddModuleScore with lists of modules genes (after conversion from mouse to human names) as an input.

To assess the uniqueness of each module we introduced the metric “signal width” which was the sum of all expression values (1 and 0.5) for a given module across all neuron types (i.e. a proxy for the number of clusters in which the module is active). The maximum signal width across all modules was 10. Then we introduced module uniqueness = 10−“signal width”.

### hdWGCNA module GO ontology

For GO ontology we used web-based service https://go.princeton.edu/cgi-bin/GOTermFinder^60^. As a background, we used a cumulative list of all 1729 genes making up all modules.

### Statistics and reproducibility

Bar graphs show the mean with actual datapoints included and error bars represent the standard error of the mean. Boxplot whiskers show maximum and minimum values, and the box's upper and lower edges define the third quartile and first quartile with the center line representing the median. All relevant statistical procedures are described in the appropriate sections of the Methods. No protocol registration was done for this study.

### Reporting summary

Further information on research design is available in the Nature Portfolio Reporting Summary linked to this article.

## Supplementary information

Supplemental Material

Reporting Summary

## Data Availability

The raw and processed sequencing data for this study have been deposited in the Gene Expression Omnibus (GEO) under the accession GSE287551. Zhang et al. data^5^ are available from GSE216039, Wang et al^3^. from GSE155622, Sharma et al.^2^ from GSE139088, Finno et al.^1^ from GSE128276, Su et al. from GSE124312, Zeisel et al.^4^ from Mousebrain.org (https://storage.googleapis.com/linnarsson-lab-loom/l1_drg.loom). Additional resources for browsing gene expression of the sensory neuronal atlas are available at: https://ernforslab.shinyapps.io/integratedDRGatlas/. All other data is available from the corresponding author upon reasonable request.

## Code availability

Links to previously published code and software used for this study are described in the relevant sections of the Methods. scCAMEL is available from: https://pypi.org/project/scCAMEL.
