# Supplementary material for: Spatial organization, chromatin accessibility and gene-regulatory programs defining mouse sensory neurons
Source: Commun Biol. 2025 Jun 11;8:908. doi: 10.1038/s42003-025-08315-1 (PMC12159162; doi:10.1038/s42003-025-08315-1)
Supplement: Supplementary file 1 — Supplemental Material [file 42003_2025_8315_MOESM1_ESM.pdf]

Supplementary Figures and Legends

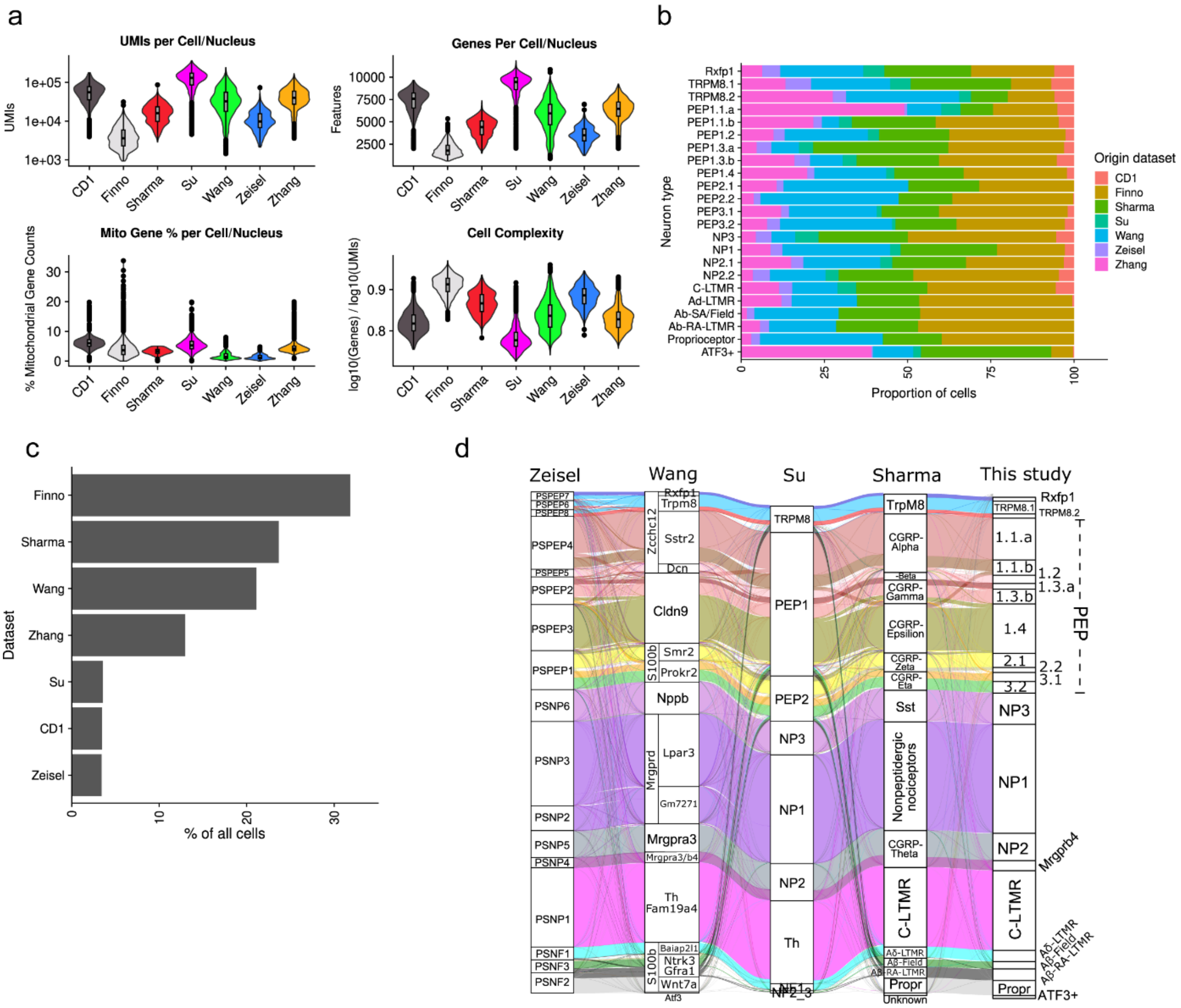

Figure S1. Construction of the integrated mouse DRG atlas. (a) Violin plots showing quality metrics for the individual datasets of the integrated atlas. (b) Percentage contribution of each original dataset to neuron types in the atlas. (c) Percentage contribution of each original dataset to the full atlas. (d) Sankey plot showing the neuron type correlations between the different original datasets as annotated in the original publications.

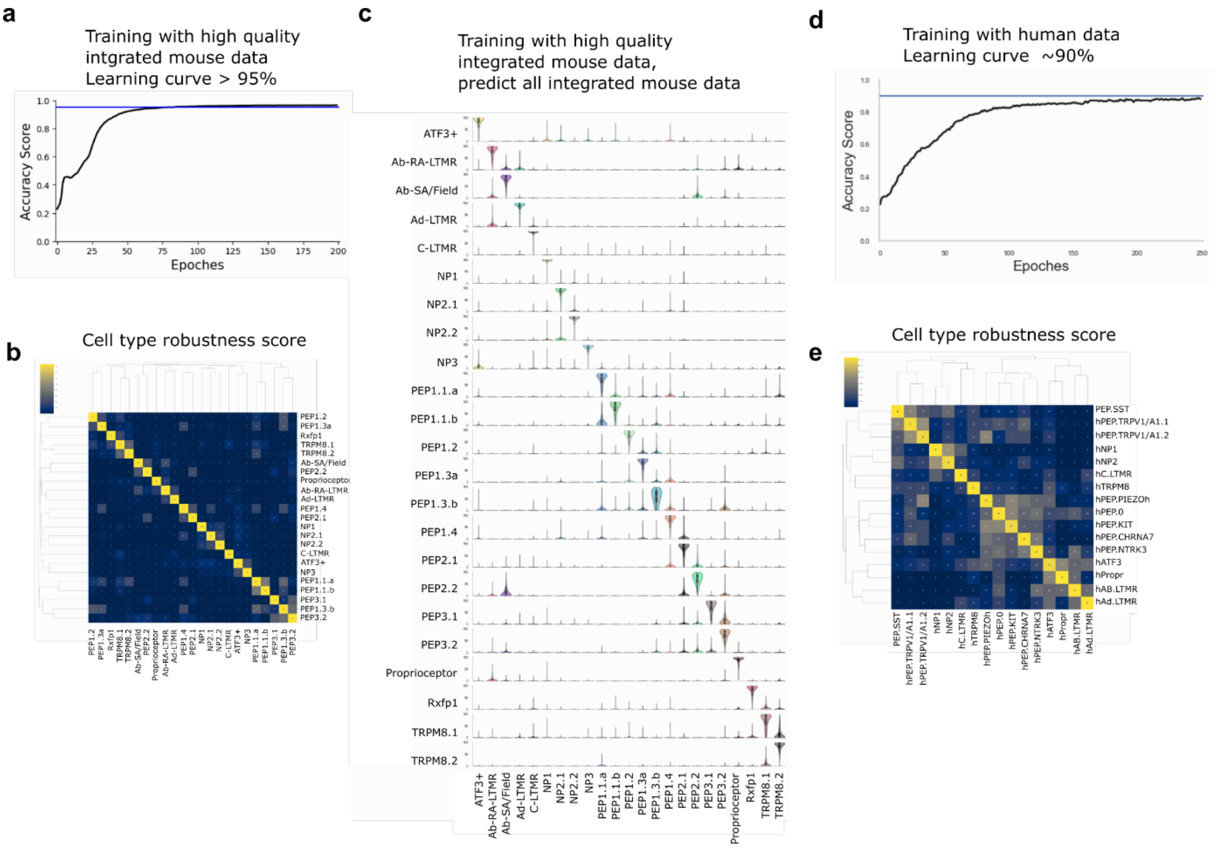

Figure S2. Metrics of machine learning of sensory neurons. (a) Learning curve representing the accuracy of the neural network classifier trained on integrated mouse neuron data. The vertical axis represents the classification accuracy, and the horizontal axis represents the number of training epochs. (b) Hierarchical heatmap showing the probabilistic similarity scores among mouse neuron cell types. Colors range from yellow (100% similarity) to dark blue (0% similarity). (c) Distribution of probability scores assigned by the neural network to each mouse neuron type, trained on high-quality integrated mouse data. (d) Learning curve representing the accuracy of the neural network classifier trained on human dorsal root ganglion (hDRG) neuron data. The vertical axis represents the classification accuracy, and the horizontal axis indicates the number of training epochs. (e) Hierarchical heatmap showing the probabilistic similarity scores among hDRG neuron cell types. Colors range from yellow (100% similarity) to dark blue (0% similarity).

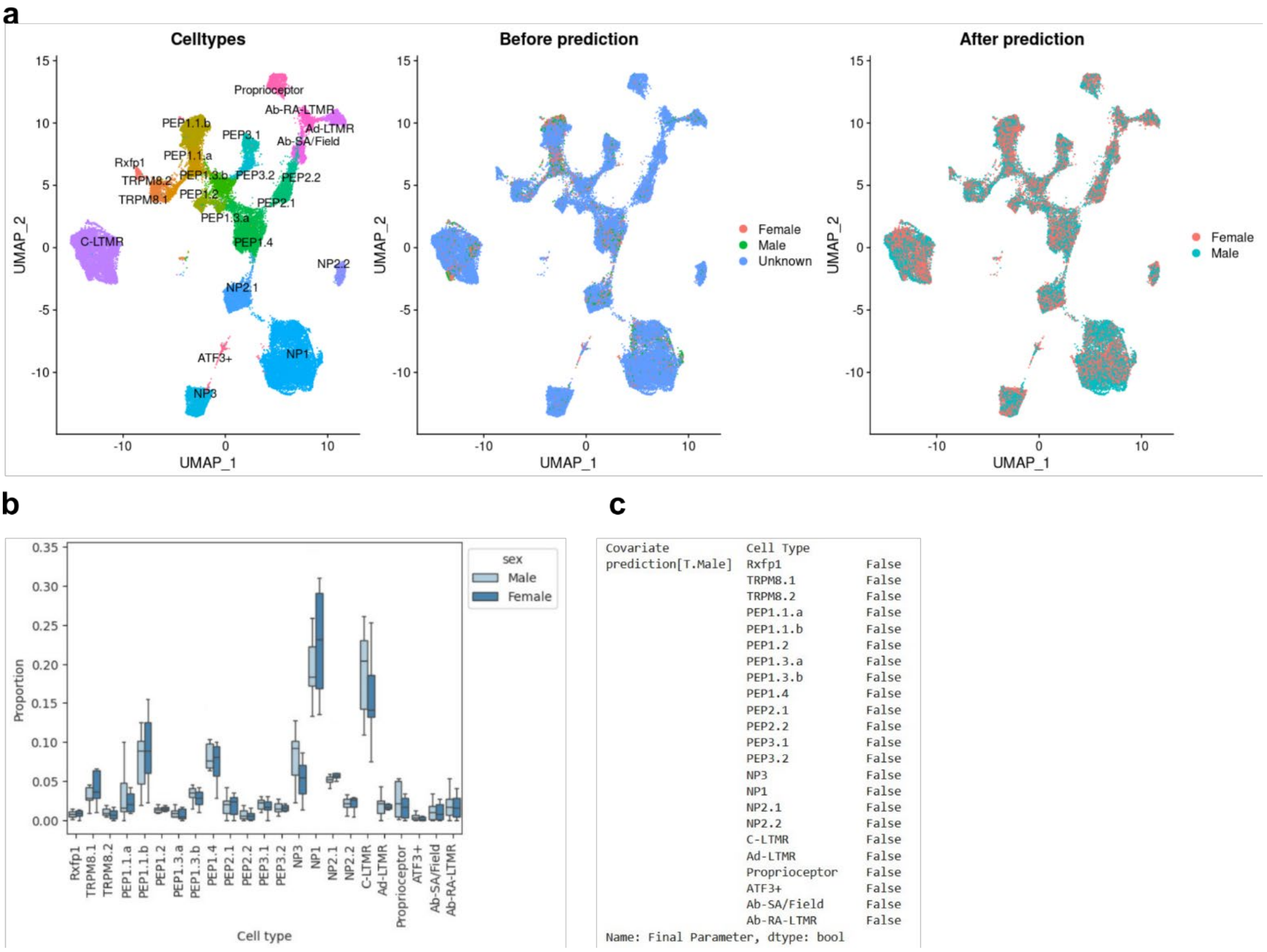


```
Covariate             Cell Type
prediction[T.Male]    Rxfp1            False
                      TRPM8.1          False
                      TRPM8.2          False
                      PEP1.1.a         False
                      PEP1.1.b         False
                      PEP1.2           False
                      PEP1.3.a         False
                      PEP1.3.b         False
                      PEP1.4           False
                      PEP2.1           False
                      PEP2.2           False
                      PEP3.1           False
                      PEP3.2           False
                      NP3              False
                      NP1              False
                      NP2.1            False
                      NP2.2            False
                      C-LTMR           False
                      Ad-LTMR          False
                      Proprioceptor    False
                      ATF3+            False
                      Ab-SA/Field      False
                      Ab-RA-LTMR       False
Name: Final Parameter, dtype: bool
```

Figure S3 Analysis of sex differences of sensory neurons. (a) (left) UMAP showing all defined cell types in the integrated atlas. (center) UMAP showing the known sex for each cell in the integrated atlas. Only few cells have known sex annotation and most are unknown. (right) UMAP showing the predicted sex of each individual cell in the dataset. (b) Boxplots showing the proportions of male and female cells for each individual cell type in the integrated atlas. (c) Output from scCODA analysis of proportional differences showing no credible differences between sexes in any cell types.

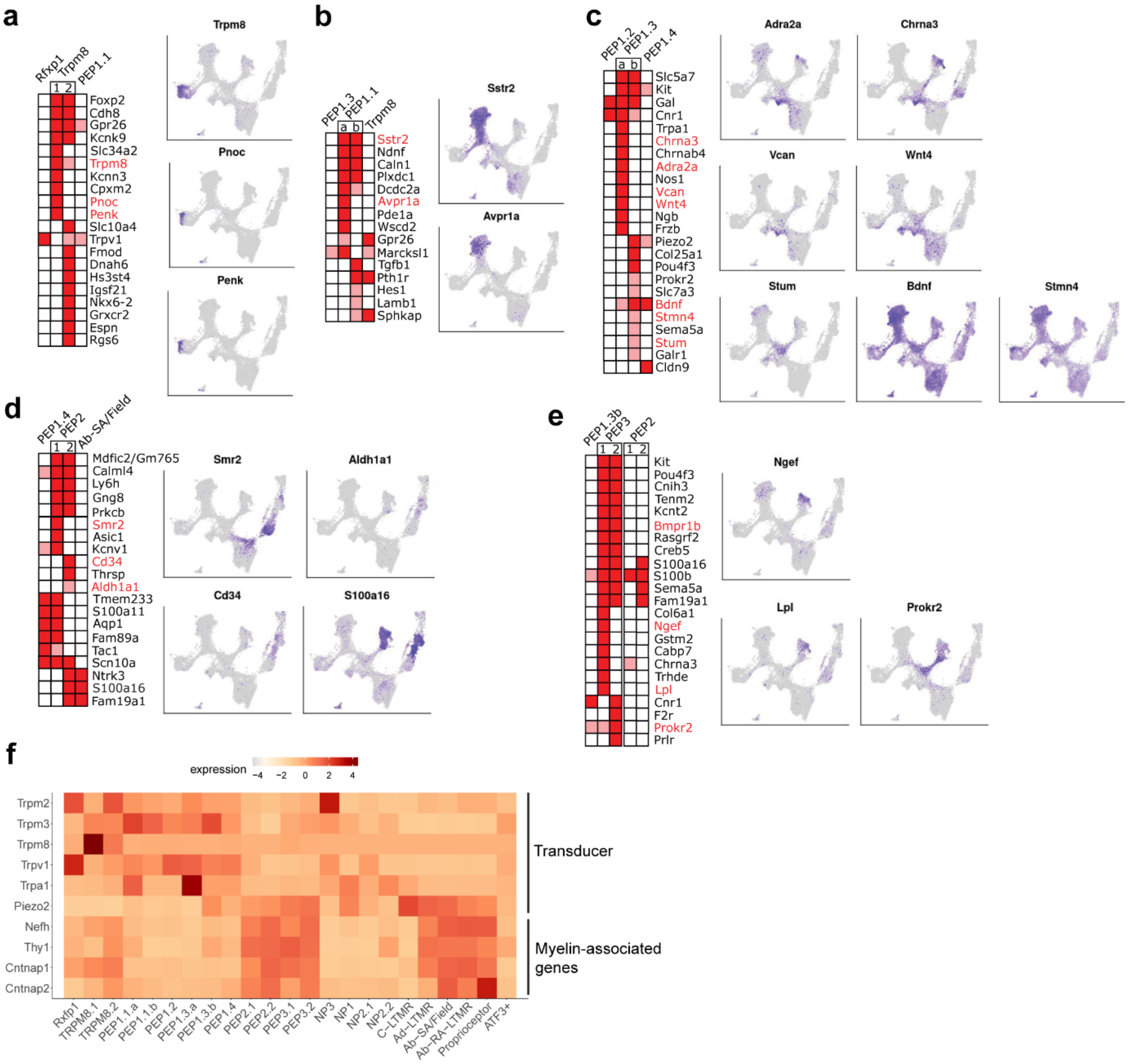

Figure S4 Neuronal cell type splits. Heatmap and marker localization in the UMAP of marker genes for (a) Trpm8, (b) PEP1.1, (c) PEP1.3, (d) PEP2 and (e) PEP3 neuronal splits. Markers used in the MERSCOPE analysis are shown in red. (f) Heatmap of transducer and myelin-associated gene expression across neuronal cell types in the integrated atlas

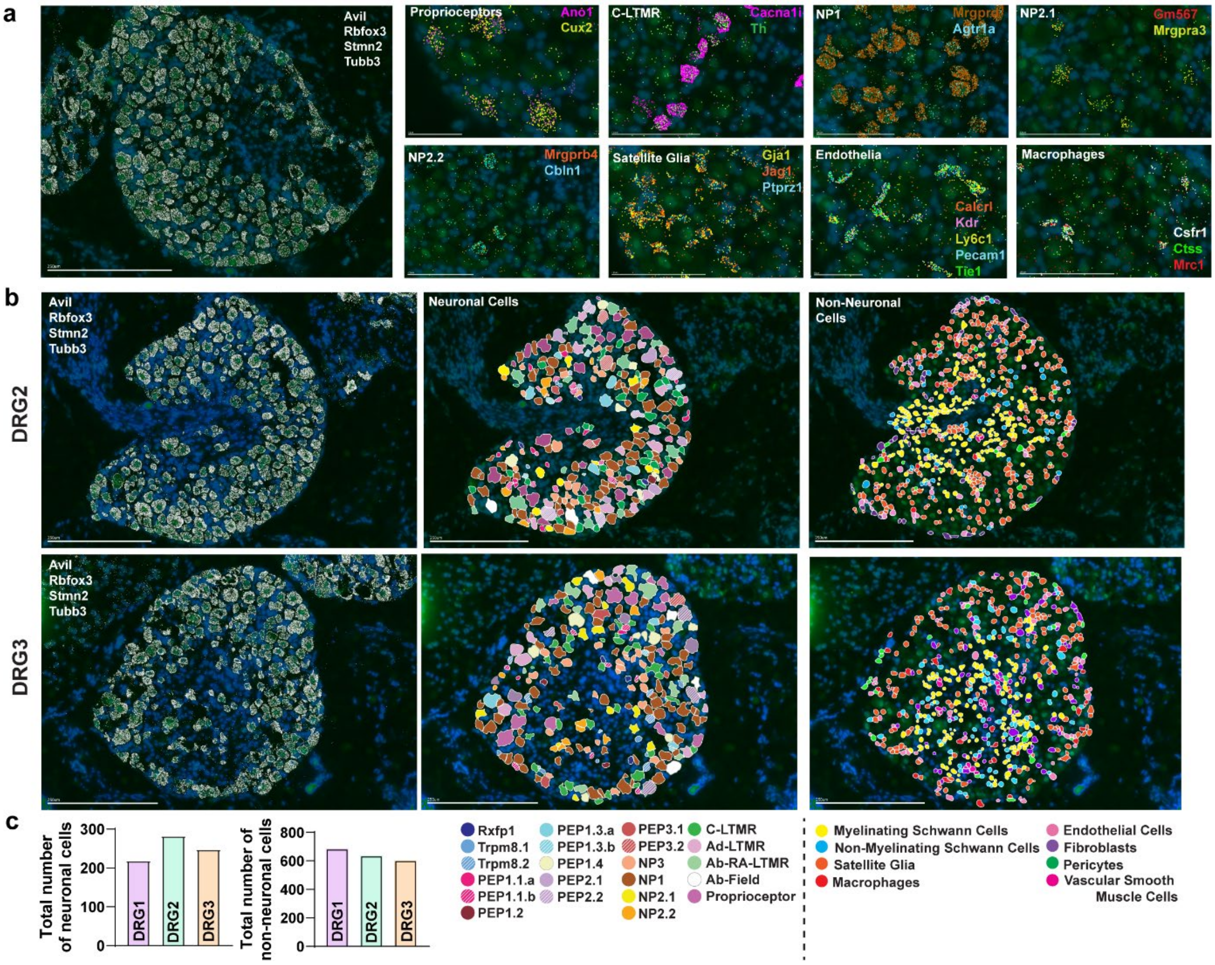

Figure S5: Extended MERSCOPE data. (a) Expression of general neuronal marker genes in the MERSCOPE analysis of DRG section one (left image, scale bar 250 µm) and images showing labeling of probes separately colored in neuronal and non-neuronal cell types in the DRG. (b) Expression of general neuronal marker genes and manual assignment of neuronal and non-neuronal cells in the MERSCOPE analysis of DRG section two (upper row) and DRG section three (lower row). Scale bar is 250 µm. (c) Quantification of the total number of neuronal (left panel) and non-neuronal (right panel) cells manually analyzed in DRG sections one, two and three.

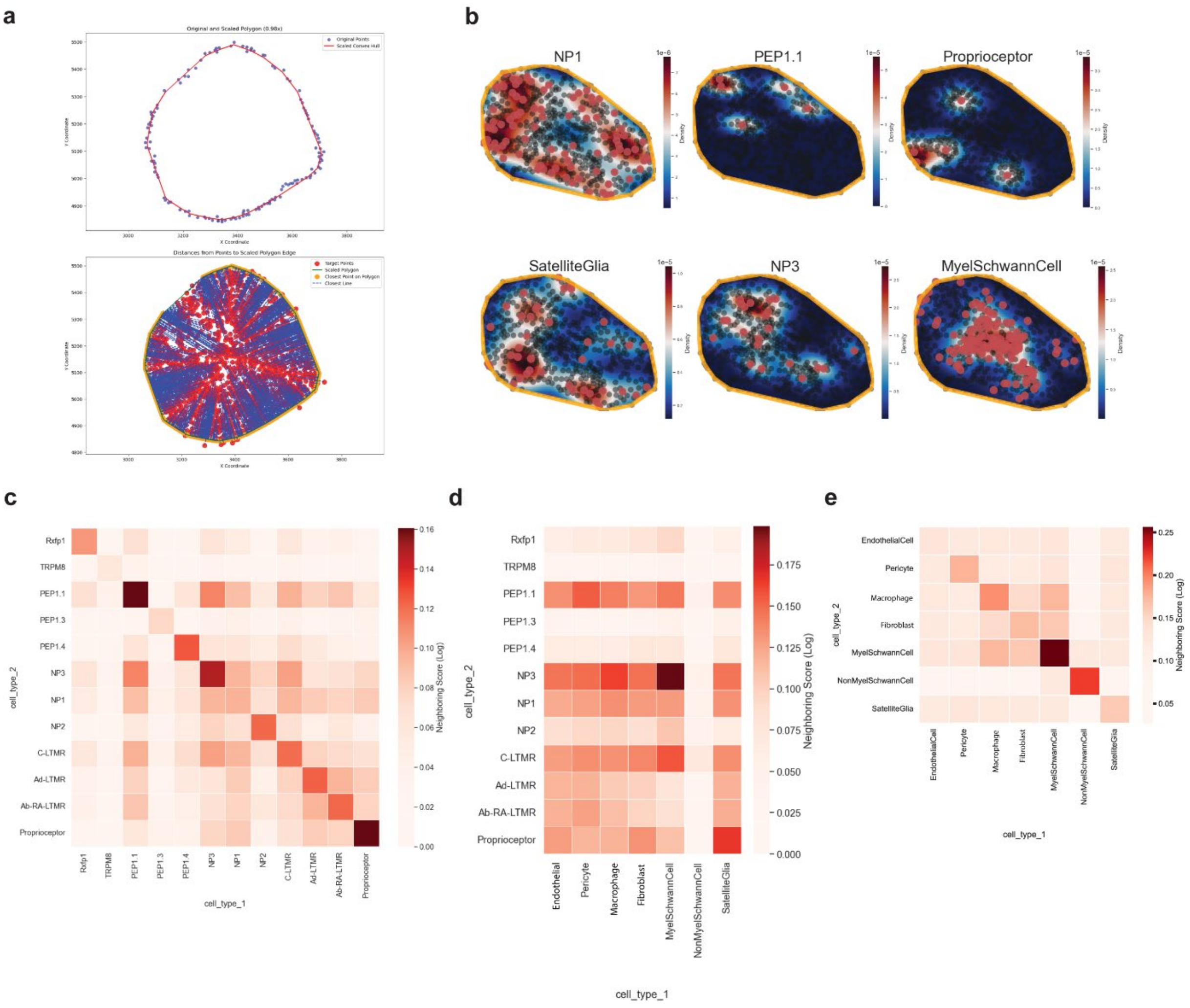

Figure S6: Spatial organization of the DRG. (a) Example image of the distance to edge quantification (Figure 3g) - for each cell (red dot) the distance to the outer polygon (upper image) was calculated. (b) Example images of Gaussian kernel density estimate of individual cell types (from left to right) NP1, PEP1.1, Proprioceptors, Satellite Glia, NP3 and myelinating Schwann cells density in a DRG section. Heatmap of scaled pairwise interaction scores between (c) neuronal vs. neuronal, (d) neuronal vs. non-neuronal and (e) non-neuronal vs. non-neuronal cell types, showing higher values for cell populations that co-localize spatially in dark red.

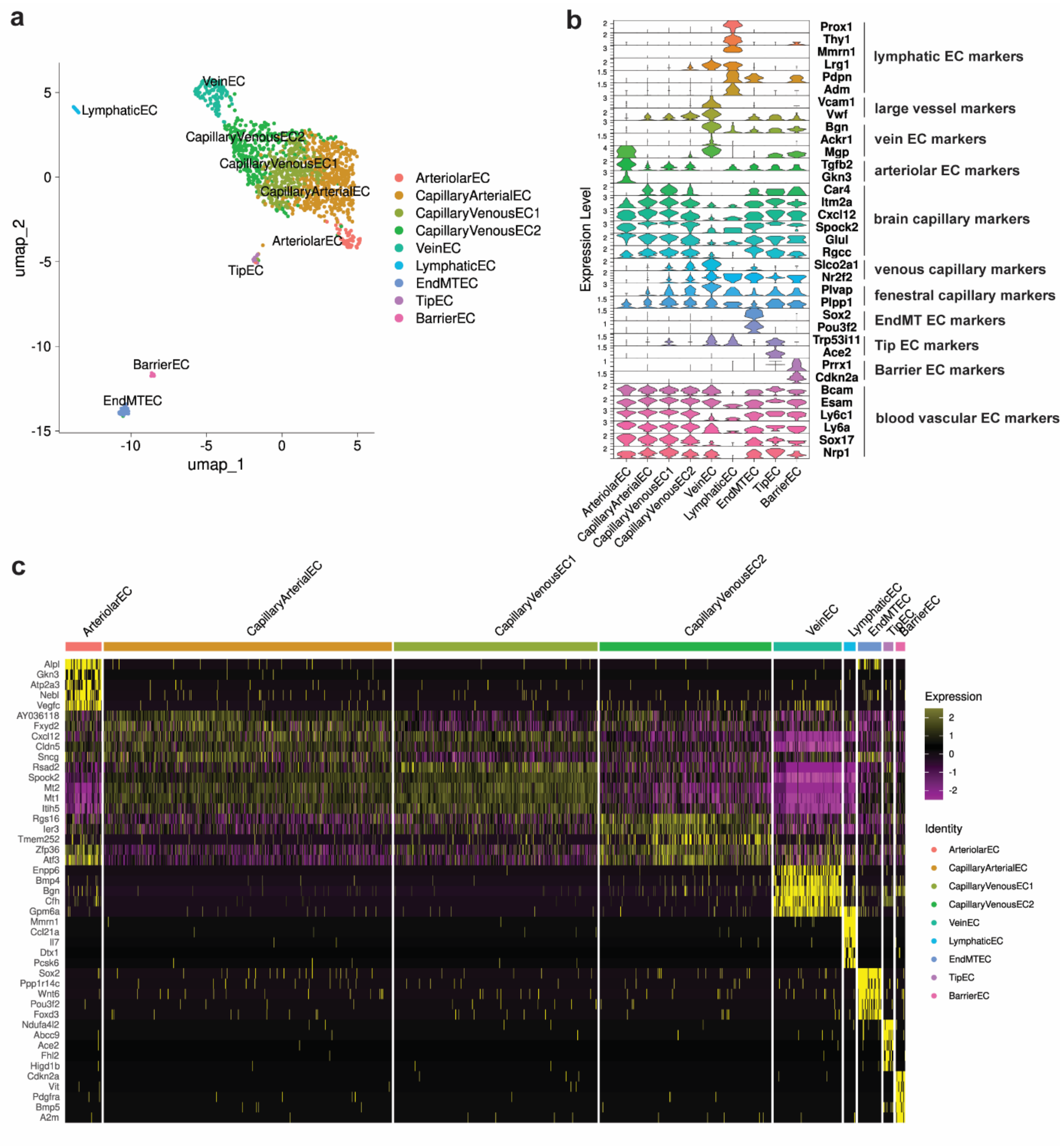

Figure S7: Endothelial cells in the DRG. (a) UMAP of endothelial cell populations in the DRG. (b) Violin plots show expression of key marker genes used to annotate subpopulations of endothelial cells. (c) Heatmap showing expression of top markers defining the subpopulations of endothelial cells.

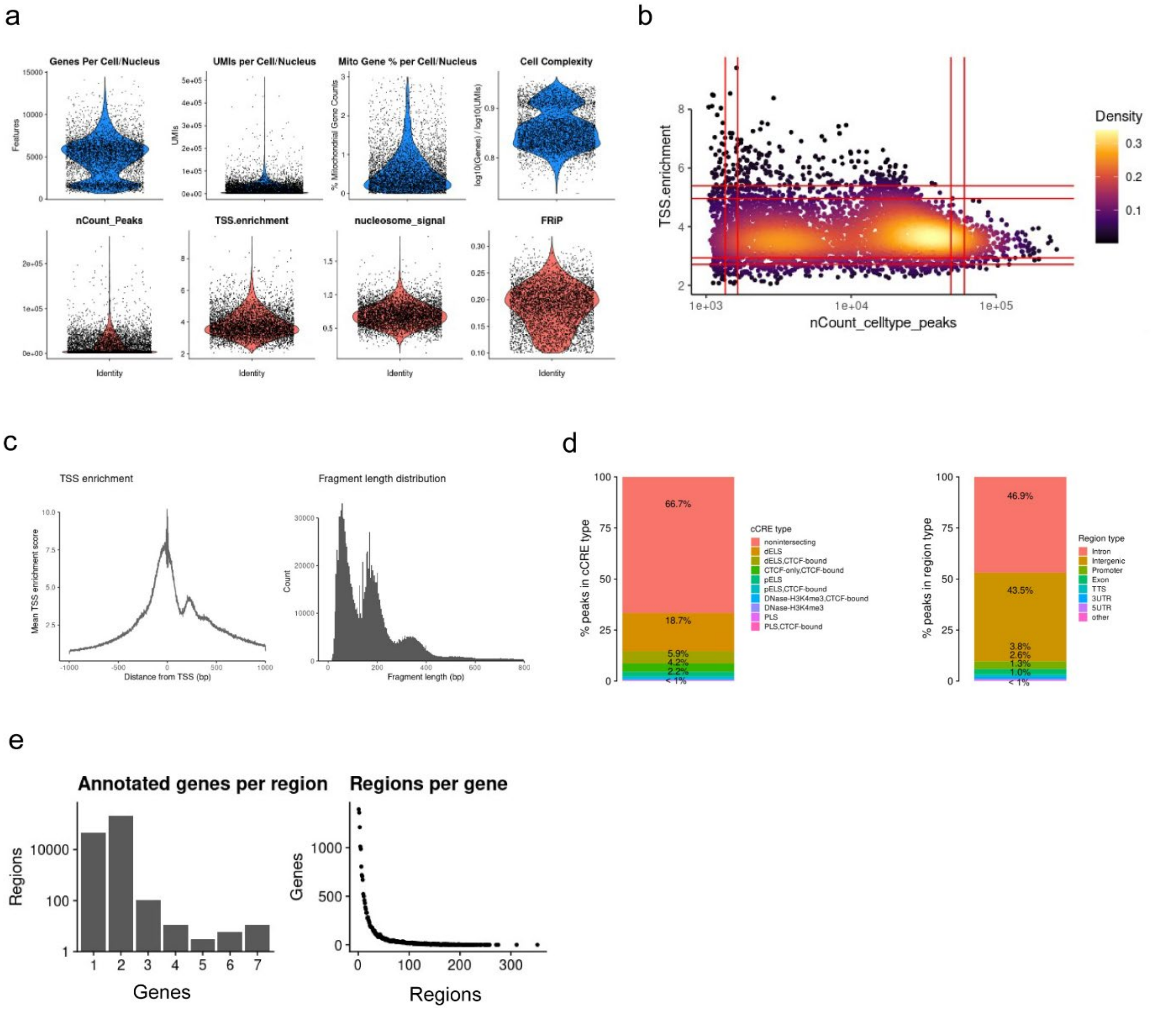

Figure S8. Mouse DRG multiome QC. (a) Quality metrics for the multiome dataset for RNA (top row) and ATAC (bottom row). (b) A Density scatterplot showing the Transcription start site (TSS) enrichment (y-axis) and fragment number (x-axis) for each nucleus in the dataset. (c) TSS enrichment distribution (left) and fragment length distribution in the ATAC data. (d) Overlap of peaks with different types of candidate cis-regulatory regions (cCREs) annotated in the ENCODE database (left). Distribution of peaks in different types of regions based on HOMER peak annotation (right). (e) Number of annotated genes for regions in the ATAC data (left). Number of regions within individual genes (right).

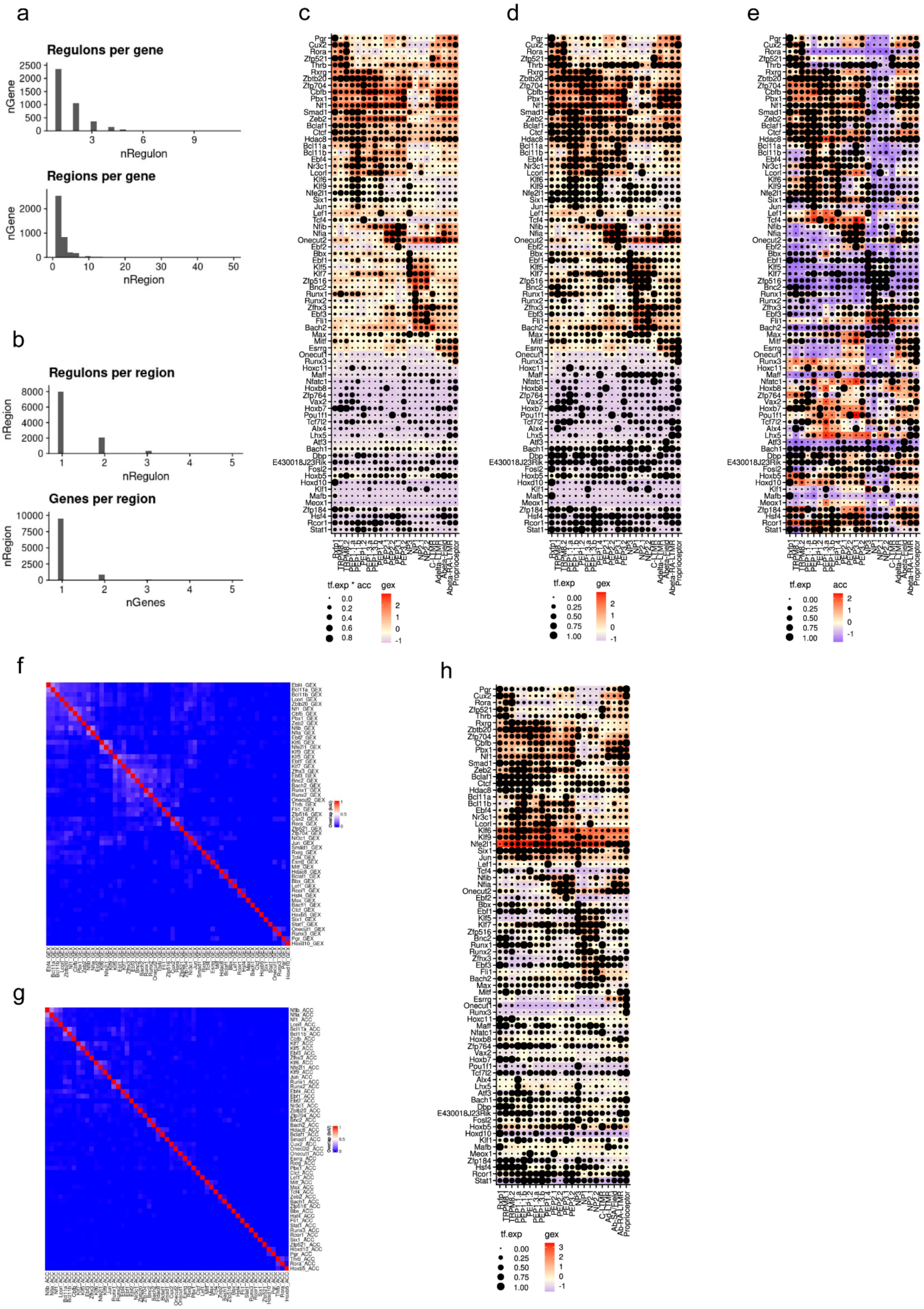

a
Regulons per gene
nGene
nRegulon
Regions per gene
nGene
nRegion
b
Regulons per region
nRegion
nRegulon
Genes per region
nRegion
nGenes
c
tf.exp * acc
gex
d
tf.exp
gex
e
tf.exp
acc
f
g
h
tf.exp
gex

Figure S9. eRegulon supplemental figure (a) Bar plots showing the number of eRegulons (x-axis) per genes (y-axis) (top). Bar plots show the number of accessible regions (x-axis) per genes (y-axis) (bottom). (b) Bar plots showing the number eRegulons (x-axis) per regions (y-axis) (top). Bar plots showing the number of genes (x-axis) controlled by regions (y-axis) (bottom). (c) Dot plot-heatmap showing the activity of all 74 eRegulons in the mouse DRG multiome data. (d) Dot plot-heatmap showing TF expression as dot size and regulon gene set activity as color. (e) Dot plot-heatmap showing TF expression as dot size and regulon region set activity as color.(f) Heatmap of the overlap of eRegulon gene modules. Color indicates Jaccard similarity. (g) Heatmap of the overlap of eRegulon region modules. Color indicates Jaccard similarity. (h) Dot plot-heatmap of the integrated atlas, showing TF expression as dot size and regulon gene set activity as color.

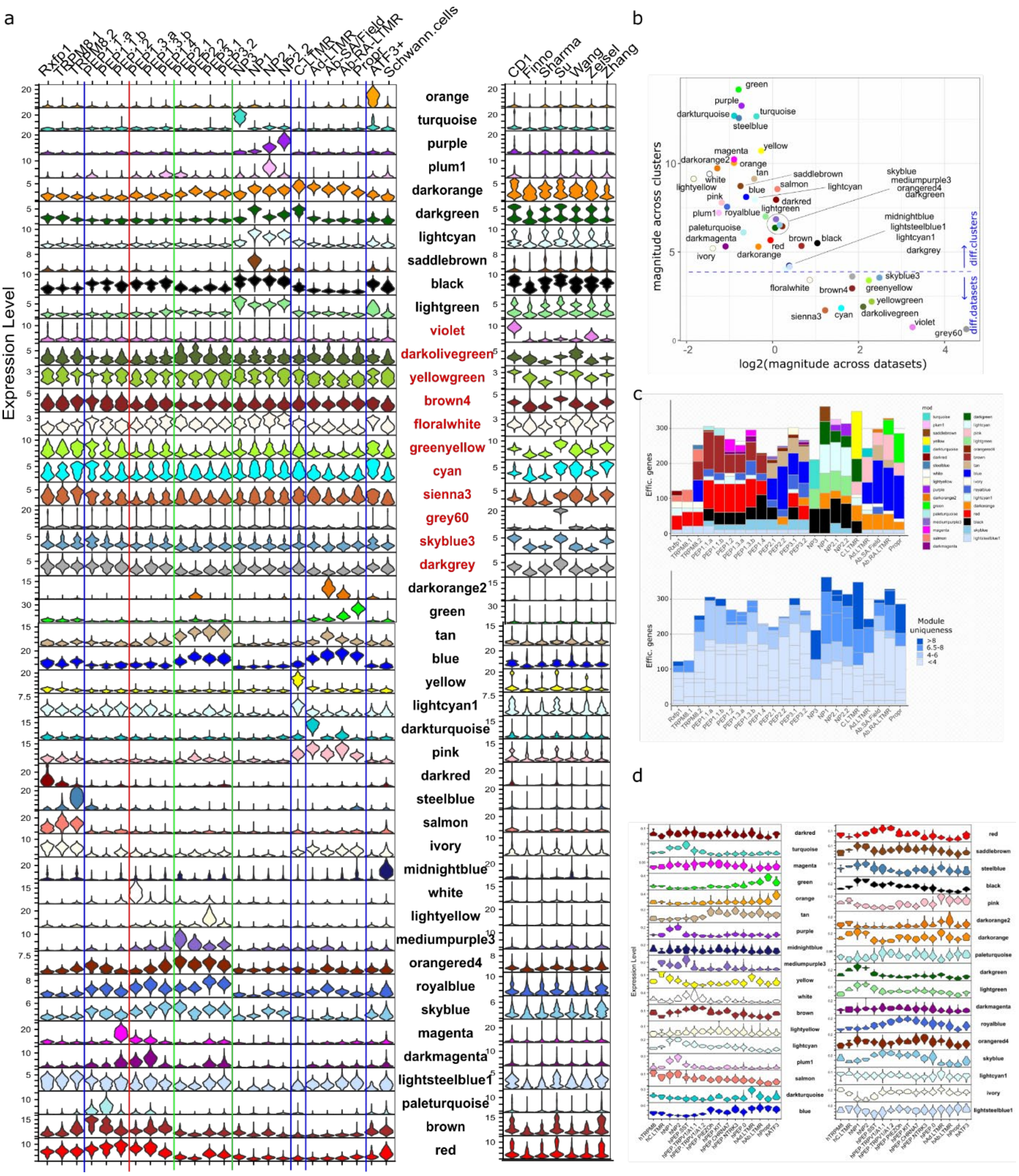

Figure S10. WGCNA Supplementary Figure (a) Violin plot of all modules across all neuronal populations and datasets. Module names in red varied across datasets more than across neuronal populations and were excluded from analysis. (b) Dot plot of module magnitudes across clusters (y-axis) and datasets (x-axis). The threshold depicted by the blue dashed line (y = 3.7) was used to separate modules differentiating neuronal types from those varying across datasets. (c) Bar plots for all module patterns across all neuronal populations (identical to Fig.6.b-e, but without separation by classes of neuronal populations). The upper plot shows module bars colored according to module names, in the lower plot bars are colored according to module uniqueness. (**d**) Mouse models projected to human dataset.

Supplementary Tables

Table S1: Atlas marker genes 349 cells per class

This Excel workbook contains marker genes for each neuron type calculated from a random sample of 349 cells of each type from the integrated atlas.

“p_val” = p-value of the marker gene for the cell type

“avg_log2FC” = average log2 fold-change for cell type against other cell types

“pct.1” = Percentage of cells expressing gene in cell type

“pct.2” = Percentage of cells expressing gene in other cell types

“p_val_adj” = Adjusted p-value for gene in cell type

“cluster” = cell type

“gene” = Gene name

Table S2: MERSCOPE gene panel list

This Excel workbook contains the 300 gene probe panel for the MERSCOPE experiment.

Table S3: Multiome Activity markers

This Excel workbook contains gene activity markers (derived from accumulated fragments in gene body and promoter region) for each DRG neuronal type in the multiome dataset

“p_val” = p-value of the marker gene for the cell type

“avg_log2FC” = average log2 fold-change for cell type against other cell types

“pct.1” = Percentage of cells with fragments in the gene in cell type

“pct.2” = Percentage of cells with fragments in the gene in other cell types

“p_val_adj” = Adjusted p-value for gene in cell type

“cluster” = cell type

“gene” = Gene name

Table S4: ChromVar

This Excel workbook contains transcription factor motif/motif family markers for each DRG neuron type calculated using ChromVar.

“p_val” = p-value of the marker motif for the cell type

“avg_diff” = average log2 fold-change for cell type against other cell types

“pct.1” = Percentage of cells with signal for motif in cell type

“pct.2” = Percentage of cells with signal for motif in other cell types

“p_val_adj” = Adjusted p-value for motif in cell type

“cluster” = cell type

“family-TF” = TF family and TF name combined

Table S5: eRegulon_metadata

This Excel workbook contains metadata for the SCENIC+ eRegulons

“Region_signature_name” = master TF and number of regions in eRegulon

“Gene_signature_name” = master TF and number of genes in eRegulon

“TF” = Name of master TF

“Region” = Regulatory region of genome controlled by TF

“Gene” = Gene controlled by TF and Region

Table S6: Atlas marker eRegulon Genes

“p_val” = p-value of the marker gene for the cell type

“avg_log2FC” = average log2 fold-change for cell type against other cell types

“pct.1” = Percentage of cells expressing gene in cell type

“pct.2” = Percentage of cells expressing gene in other cell types

“p_val_adj” = Adjusted p-value for gene in cell type

“cluster” = cell type

“gene” = Gene name

“eReg” = Name of eRegulon master TF; NA if gene is not in any eRegulon

“eReg_gene” = TRUE if gene belongs to any eRegulon

“gex” = eRegulon gene set activity score; NA if gene is not in any eRegulon

“acc” = eRegulon region set activity score; NA if gene is not in any eRegulon

“tf.avg.exp” = Average expression of eRegulon TF in cell type; NA if gene is not in any eRegulon

“tf.pct.exp” = Percentage of cells in cell type expressing eRegulon TF; NA if gene is not in any eRegulon

Table S7: Genes_for_46_modules

This Excel workbook contains four sheets:

1. ”Legend”: description of all other sheets.

2. ”Genes_for_35_mod_diff_CLUST”: Lists of genes making up 35 modules which contrast CLUSTERS.

3. ”Genes_for_11_mod_diff_ DATASETS”: Lists of genes making up 11 modules which contrast DATASETS.

4. ”Genes_with_R-corr”: list of all genes making up all modules with additional info:

Column ”mod”. Module name to which given row relates

Column ”R”. Pearson correlation coefficient of given gene to the eigengene of given module

Column ”status”: specifies if given gene is uniquely assigned to given module (”unique”). If assignment is not unique, it column specifies if the gene has highest (or second highest) R corr to given module (”1st” or ”2nd”).

Table S8: GO_for_modules

This Excel workbook contains nine sheets grouped by type of term filtering. In each group there are three tabs, one for each GOtype: Biological Process (BP), Molecular Function (MF) and Cellular Component (CC). Groups are:

”_full” (tab color – grey) – all GO terms for all modules

”_clust” (tab color – green) – all GO terms only for modules contrasting clusters (not datasets)

”_clean” (tab color – purple) – relevant manually chosen GO terms for modules contrasting clusters (not datasets)

Majority of columns in each tab is self-explanatory (output from web based GO ontology engine except:

Column ”contrast”: specifies if given module contrast clusters (neuron types) or datasets

Column ”Manually picked”: specifies with ”*” terms which are chosen from tabs ”_clust” to be preserved in tabs ”_clean”.
